# Role of BDNF–TrkB signaling in the antidepressant‐like actions of loganin, the main active compound of Corni Fructus

**DOI:** 10.1111/cns.14305

**Published:** 2023-07-05

**Authors:** Mingzhu Gong, Junming Wang, Lingling Song, Xiaohui Wu, Yanmei Wang, Bingyin Li, Yueyue Zhang, Lingyu Qin, Yaqian Duan, Bingyu Long

**Affiliations:** ^1^ College of Pharmacy Henan University of Chinese Medicine Zhengzhou China; ^2^ Co‐Construction Collaborative Innovation Center for Chinese Medicine and Respiratory Diseases by Henan & Education Ministry of P.R. China Henan University of Chinese Medicine Zhengzhou China

**Keywords:** antidepressant, BDNF–TrkB signaling, chronic unpredictable mild stress, Corni Fructus, loganin

## Abstract

**Aims:**

Corni Fructus (CF) and some CF‐contained prescriptions are commonly used in clinical treatment of depression. This investigation aims to evaluate the main active compound of CF in antidepressant properties and its key target.

**Methods:**

Firstly, this study established a behavioral despair model and used high‐performance liquid chromatography method to evaluate the antidepressant‐like effects of water extract, 20%, 50%, and 80% ethanol extracts of CF, and its main active compound. Then, this study created chronic unpredictable mild stress (CUMS) model to assess loganin's antidepressant‐like properties, and its target was evaluated by quantitative real‐time polymerase chain reaction, Western blot, Immunofluorescence, enzyme‐linked immunosorbent assay, and tyrosine receptor kinase B (TrkB) inhibitor.

**Results:**

Results showed that the different extracts of CF significantly shortened the immobility time in forced swimming and tail suspension tests. Moreover, loganin alleviated CUMS‐induced depression‐like behavior, promoted neurotrophy and neurogenesis, and inhibited neuroinflammation. Furthermore, K252a blocked the improvement of loganin on depression‐like behavior, and eliminated the enhancement of neurotrophy and neurogenesis and the inhibition of neuroinflammation.

**Conclusion:**

Overall, these results indicated that loganin could be used as a major active compound of CF for the antidepressant‐like properties and exerted antidepressant‐like actions by regulating brain derived neurotrophic factor (BDNF)–TrkB signaling, and TrkB could be used as key target for itsantidepressant‐like actions.

## Graphical abstract

Loganin, as a main active compound of CF, exerts antidepressant‐like actions by regulating BDNF–TrkB signaling.

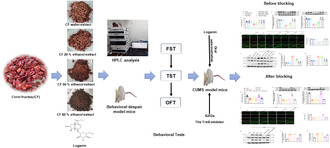

## 1 INTRODUCTION

Depression is a psychiatric disorder with is high morbidity rate, high recurrence rate, and high disability rate.^1^, ^2^ However, novel and effective antidepressants with clear components, clear mechanisms, and explicit targets are currently lacking. In recent years, multiple lines of evidence suggested that tyrosine receptor kinase B (TrkB) target was crucial in the pathogenesis of depression.^3^ For example, clinical autopsy analyses found that patients with depression have lower level of TrkB in brain, whereas patients treated with some antidepressants (e.g., fluoxetine) have higher level of TrkB.^4^, ^5^ For another, preclinical results showed that the level of TrkB in the hippocampus of depression was low, which was reversed after the treatment of antidepressants (such as iridoid glycoside compound geniposide).^6^, ^7^ TrkB was a specific binding site for brain derived neurotrophic factor (BDNF) and BDNF was considered a potential neurobiological marker for depression.^8^, ^9^ Clinical autopsy data shown that decreased levels of BDNF and increased levels of proBDNF in brain samples from depressed patients.^10^ These evidences point to an important hot spot for the development of highly effective antidepressants with a clear mechanism around the TrkB target. Therefore, the TrkB target undoubtedly play a positive role in revealing the pathogenesis of depression.

Corni Fructus (CF) is a traditional Chinese medicine commonly used in clinical treatment of central nervous system, and has a wide range of biological activities (including neuroprotection, immune enhancement, anti‐inflammatory, etc.).^11^ In particular, CF and its important formulations (such as Liuwei Dihuang Decoction and Zuogui Jiangtang Jieyu Decoction) were outstanding in clinical treatment of depression.^12^, ^13^ However, to date, the major active compound of CF's antidepressant‐like actions and its target are still unknown.

This study first used the high‐performance liquid chromatography (HPLC) method to observe the content of loganin in water and ethanol extracts of CF, and behavioral despair model was used to evaluate the antidepressant efficacy of different extracts.^14^, ^15^ We further conducted a correlation analysis between the content and antidepressant efficacy to determine the main active compound of CF in antidepressant therapy. In addition, numerous studies demonstrated that K252a was a recognized and commonly used TrkB inhibitor.^16^, ^17^ In the present study, after determining the main antidepressant active compounds of CF, this study futher used K252a to explore its key antidepressant targets. It is also considered that neurotrophy, neurogenesis, and neuroinflammation were often affected by activation of TrkB targets, and were critical in the pathogenesis of depression.^18^, ^19^ Based on this, the important factors related TrkB were used in this study to explore its antidepressant‐like mechanisms. The above research design aims to reveal the main active compounds of CF, as well as its targets and mechanisms for antidepressant‐like actions, which provide preclinical experimental evidence for more safe, scientific and effective prevention and treatment of depression.

## 2 MATERIALS AND METHODS

### 2.1 Experimental animals

Adult ICR male (3–4 weeks old) mice with the body weight of 18–23 g were purchased from the Experimental Animal Center of Henan Province [Zhengzhou, China; certificate no. SCXK (Yu) 2017‐0001] and allowed to acclimate the experiment environment for 7 days before any procedure. The mice were fed under a light/dark cycle (12:12 h, 7 am/7 pm) and temperature (23 ± 1°C). All the procedures were strictly followed the People's Republic of China's legislation on the use and care of laboratory animals and guidelines formulated by the Institute for Experimental Animals of Henan University of Chinese Medicine and were approved by the Experimental Animal Ethics Committee of Henan University of Chinese Medicine (the approval number DWLL201903531).

### 2.2 Preparation of CF extracts

Refer to the Pharmacopeia of the People's Republic of China (2020 Edition) for proper CF to be weighed. Firstly, an equal amount of CF powder was weighed and soaked in water, 20% ethanol, 50% ethanol, and 80% ethanol for 1 h, respectively. Next, the traditional reflux extraction method was used to extract two times, and the filtrate was concentrated and then freeze‐dried to obtain the freeze‐dried powder of the extract of different solvents or concentrations of CF. Also, under the conditions that the mobile phase was 1% phosphoric acid and acetonitrile (12:88), the column temperature was 30°C, the flow rate is 1 mL/min and the detection wavelength was 240 nm, the content of loganin in different CF extracts was determined by HPLC.

### 2.3 Drugs and treatment

**FIGURE 1:**
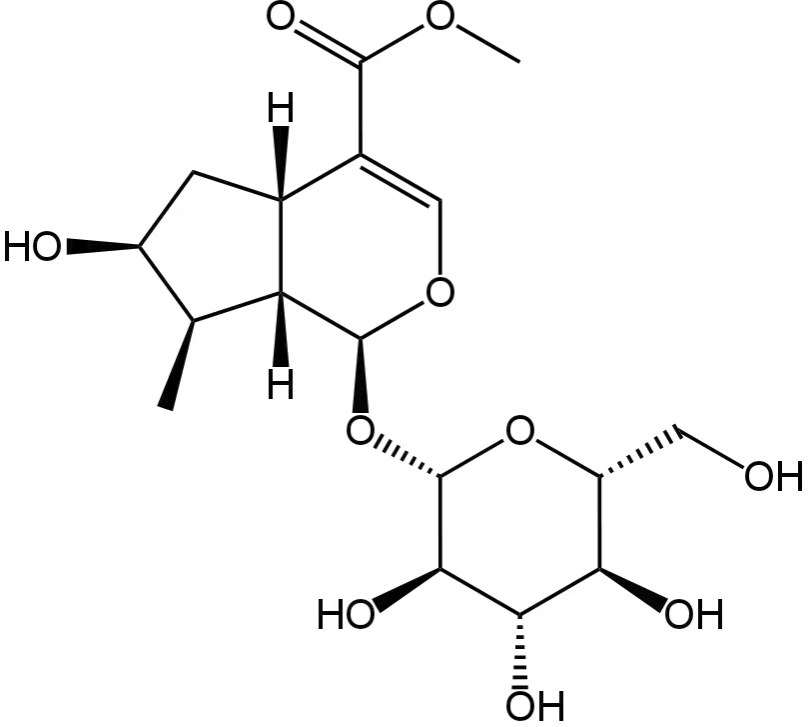
The chemical structure of loganin (Chemical formula: C_17_H_26_O_10_; molecular weight: 390.3823).

CF was purchased from Anhui Xiehecheng Pharmaceutical Decoction Piece Co., Ltd. (Anhui, China). Numerous studies showed that loganin has many biological activities related to central nervous system diseases, such as anti‐Alzheimer's disease, anti‐Parkinson's disease, sedation and hypnosis, and has been proved to pass through the blood–brain barrier.^20^, ^21^, ^22^ Loganin (its chemical structure in Figure 1) and fluoxetine hydrochloride (FH) were both brought from Shanghai Yuanye Bio‐Technology Co., Ltd. (Shanghai, China), and the purity was above 98% as determined by HPLC. K252a was brought from Beijing Solarbio Science & Technology Co., Ltd. (Beijing, China).

**FIGURE 2:**
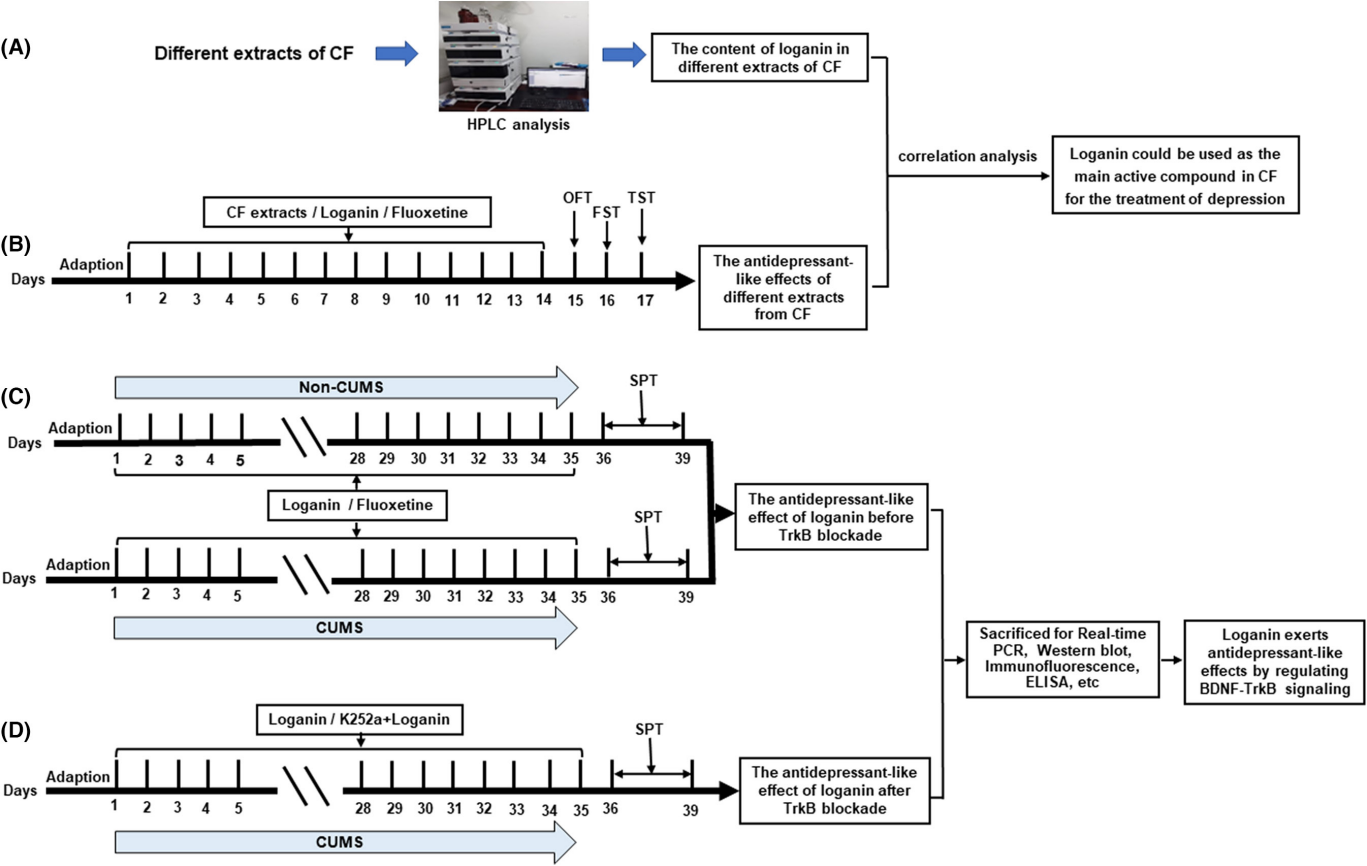
A schematic diagram of our experimental design. A detailed experimental schedule for detecting the content of loganin in different extracts of CF (A). A detailed experimental schedule for studying the antidepressant‐like activity of different extracts of CF (B). A detailed experimental schedule for studying the antidepressant‐like actions of loganin before TrkB blockade (C). A detailed experimental schedule for studying the antidepressant‐like actions of loganin after TrkB blockade (D).

Firstly, we performed the first animal experiment to evaluate the antidepressant‐like actions of different extracts of CF and loganin in mice. According to the experimental grouping, mice were orally administered with saline (NaCl 0.9%)/different extracts of CF (640 mg/kg)/loganin (10 mg/kg)/FH (10 mg/kg) daily for 14 consecutive days. Next, we conducted the second animal experiment using the chronic unpredictable mild stress (CUMS) model to explore the antidepressant‐like actions of loganin in mice. According to the experimental grouping, mice were exposed to CUMS and were administrated NaCl 0.9%/loganin (5, 10, or 20 mg/kg)/FH (10 mg/kg) for 35 consecutive days. Finally, we performed the third experiment to determine whether the TrkB target was essential for the antidepressant‐like actions of loganin. According to the experimental grouping, mice were subjected to CUMS and received a daily oral administration of NaCl 0.9%/loganin (20 mg/kg)/FH (10 mg/kg), as well as a daily intraperitoneal injection of K252a/DMSO for 35 consecutive days. The entire experimental design is shown in Figure 2.

### 2.4 CUMS procedure

The CUMS procedure refers to previous reports.^23^ The specific operations of the CUMS program were as follows: 5 min cold swim at 4 ± 2°C, 24 h food deprivation, 5 min warm swim at 45 ± 2°C, 24 h water deprivation, 24 h soiled cage, 3 h ultrasound, 1 min tail pinch, 10 min cage shaking. Mice were randomly exposed to one of the stressors every day for 35 days.

### 2.5 Behavioral tests

#### 2.5.1 Open field test (OFT)

OFT was performed as previously described.^24^ The mice were placed at the bottom of the all‐black experimental device (50 × 50 × 40 cm) and allowed to move freely for 5 min. Two well‐trained observers recorded the crossing (considered as the horizontal score) and standing (considered as the vertical score) for 5 min.

#### 2.5.2 Forced swimming test (FST)

FST was based on the existing reports and slightly modified.^25^ Mice were placed individually into a glass container (height: 30 cm, diameter: 15 cm) with water at a temperature of 24 ± 2°C. The test lasted for 6 min, and the observer recorded the immobility time for the last 4 min.

#### 2.5.3 Tail suspension test (TST)

TST was slightly modified based on existing literature reports.^26^ Each mouse was suspended 50 cm from the ground with tape. The test lasted for 6 min, and the observer recorded the immobility time for the last 4 min.

#### 2.5.4 Sucrose preference test (SPT)

SPT was conducted with reference to the previous research methods.^27^ In brief, two bottles of 1% sucrose solution were placed in each mouse cage for 24 h, and then one bottle of sucrose solution was replaced by pure water for 24 h. After adaptation, mice were deprived of water and food for 24 h. During the formal test, sucrose solution and water were placed in the cage for 24 h. Two bottles were weighed at the end of the test. Sucrose preference (%) = sucrose intake (g)/[sucrose intake (g) + pure water intake (g)] × 100%.

### 2.6 Tissue sample collection

The hippocampus of each mouse was quickly removed on the ice platform after sacrifice and then stored in a freezer set to maintain −80°C until analysis.

### 2.7 Quantitative real‐time polymerase chain reaction

Total RNA was extracted with TRIzol reagent and reverse transcribed into cDNA by PrimeScript RT reagent Kit according to the manufacturer's instructions. Relative mRNA expression levels were performed with the SYBR Green detection system. All samples were performed normalized with β‐actin mRNA level. The sequences of primers are shown in Table 1.

**TABLE 1:** Primer sequences.

Gene	Primer sequences
β‐Actin	Forward: GTGACGTTGACATCCGTAAAGA
	Reverse: GTAACAGTCCGCCTAGAAGCAC
TrkB	Forward: ATCACCAACAGTCAGCTCAAGC
	Reverse: TTCAGCGTCTTCACAGCCAC
BDNF	Forward: TATTAGCGAGTGGGTCACAGCG
	Reverse: TACGATTGGGTAGTTCGGCATT
PI3K	Forward: CACGGCGATTACACTCTTACACTA
	Reverse: CACTGGGTAGAGCAACTTCACATC
Akt	Forward: CTTCCTCCTCAAGAACGATGGC
	Reverse: TGTCTTCATCAGCTGGCATTGT
NF‐κB	Forward: CGAGTCTCCATGCAGCTACG
	Reverse: TTTCGGGTAGGCACAGCAATA
NLRP3	Forward: TAAGAACTGTCATAGGGTCAAAACG
	Reverse: GTCTGGAAGAACAGGCAACATG

### 2.8 Western blot analysis

The total protein was obtained from the hippocampus of 5 mice in each group.^17^, ^28^, ^29^ The protein samples were separated by SDS‐PAGE and then transferred to PVDF. Use one of the following antibodies such as anti‐nuclear factor‐κB (NF‐κB) (1:1000), anti‐phosphorylated (p)‐NF‐κB (1:1000), anti‐nod‐like receptor family pyrin domain containing 3 (NLRP3) (1:1000), anti‐phosphoinositide 3‐kinase (PI3K) (1:1000), anti‐p‐PI3K (1:1000), anti‐protein kinase B(Akt) (1:1000), anti‐p‐Akt (1:1000), anti‐BDNF (1:1500), anti‐TrkB (1:1000), anti‐p‐TrkB (1:1000), β‐actin (1:2500) from Gene Tex. Membranes were incubated with primary antibodies overnight at 4°C followed by secondary antibodies. Then Tanon imaging system was detected to visualize the bands. Finally, the Image Lab 3.0 system tool was used for densitometry. In the current study, the Western blot method was used to detect indicators closely related to neurotrophy and neuroinflammation (Appendix S1).

### 2.9 Enzyme‐linked immunosorbent assay

The respective kits were purchased from the Jiangsu Enzyme‐free Industry Co., Ltd (Jiangsu, China), and the levels of tumor necrosis factor‐alpha (TNF‐α) and interleukin‐1β (IL‐1β) were detected in the collected hippocampal tissues according to the kits instructions.

### 2.10 Immunofluorescence

After the mice were sacrificed, their brains were fixed with 4% paraformaldehyde. For the neuron nucleus/bromodeoxyuridine (NeuN^+^/DCX^+^) double labeling experiment, the primary antibody was added to the slices and incubated overnight at 4°C. The next day, the slices were washed and dried, the secondary antibody HRP Rabbit anti‐goat and anti‐fluorescence quenching sealing agent were added dropwise in the circle. Finally, the sections were analyzed under a fluorescence microscope.

### 2.11 Statistical analysis

All experimental data were expressed as the mean ± standard deviation (SD). Statistical analysis was performed using the Statistics Package for Social Science program version 25.0 (SPSS, Chicago, IL, USA). We used the Shapiro–Wilk test to assess the normality of the distribution of continuous variables, and among multiple groups determined by the one‐way analysis of variance (ANOVA). All data have been tested for normality, and data that do not exhibit a normal/Gaussian distribution be analyzed via a non‐parametric equivalent. Values of *p* < 0.05 were considered statistically significant. GraphPad Prism 8.0 was used for drawing graphs.

## 3 RESULTS

### 3.1 Loganin could be used as an important active compound of CF for antidepressant‐like actions

#### 3.1.1 The content of loganin in different extracts of CF

**FIGURE 3:**
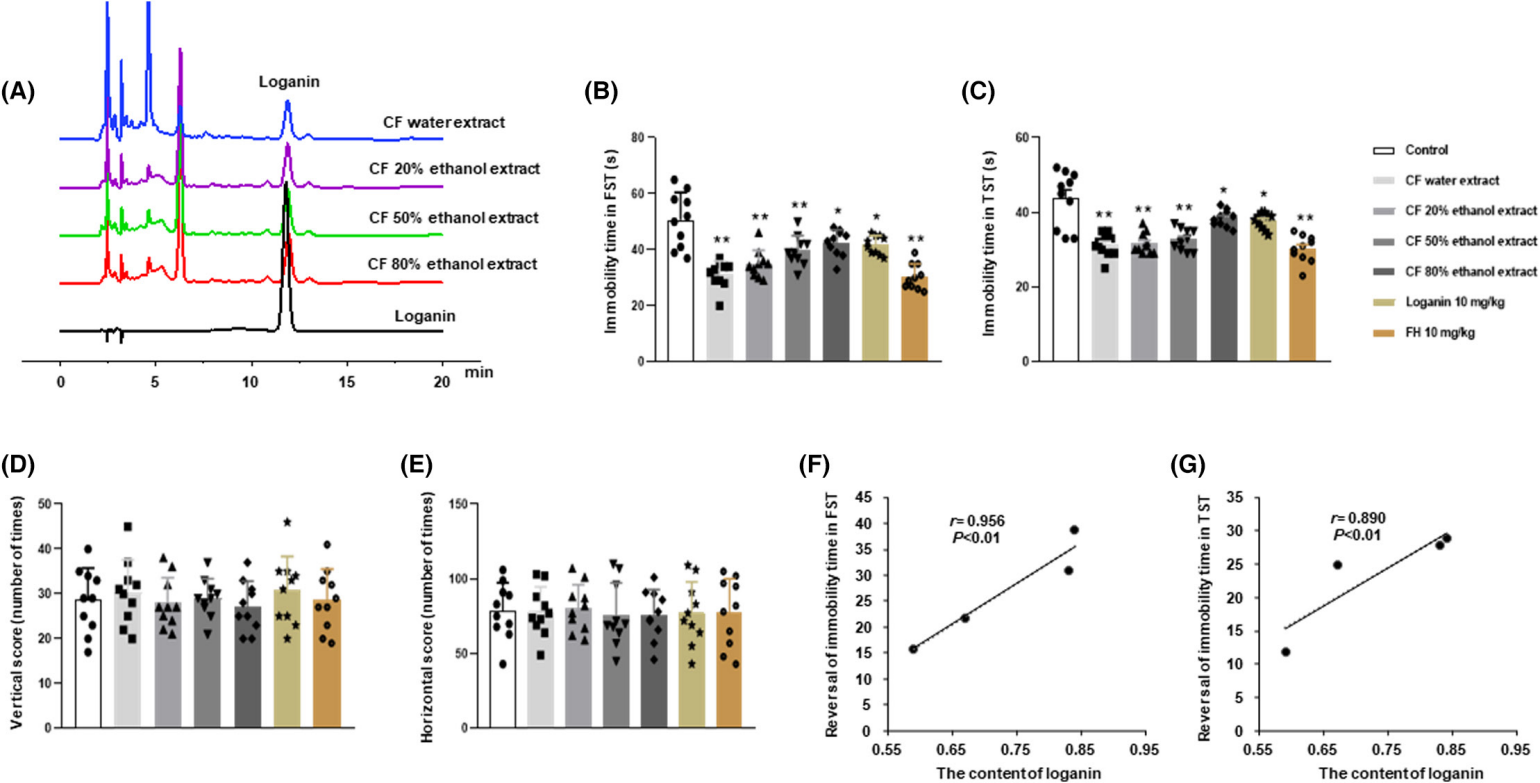
Loganin could be used as an important active compound of CF for antidepressant‐like actions. HPLC chromatograms of loganin in different extracts of CF (A), impact of different extracts of CF on the swimming immobility time in FST [*F*_(6, 63)_ = 15.702; *p* < 0.01, *p* < 0.01, *p* < 0.01, *p* < 0.05, *p* < 0.05, and *p* < 0.01] (B), and the suspension immobility time in TST [*F*_(6, 63)_ = 16.691; *p* < 0.01, *p* < 0.01, *p* < 0.01, *p* < 0.05, *p* < 0.05, and *p* < 0.01] (C), horizontal scores [*F*_(6, 63)_ = 0.370; *p* = 0.996, *p* = 1, *p* = 1, *p* = 0.999, *p* = 0.988, and *p* = 1] (D), and vertical scores [*F*_(6, 63)_ = 0.068; all *p* = 1] (E) in OFT, and the correlation analysis between the reversal rate of immobility time of different extracts of CF in FST (F) and TST and content of loganin (G). Data were shown as mean ± SD (*n* = 10). *p < 0.05 and **p < 0.01 compared with the control group.

Firstly, the content of the main active compound loganin in different extracts of CF was detected by HPLC, and the chromatogram is shown in Figure 3A. In the present study, the contents of water extract, 20%, 50%, and 80% ethanol extracts of CF were 0.84%, 0.83%, 0.67%, and 0.59%, respectively, among which the water extract of CF contained the highest content of loganin.

#### 3.1.2 The different extracts of CF have antidepressant‐like actions

This study evaluated the antidepressant‐like actions of different extracts of CF and its main active compound loganin by the behavioral despair model. The results revealed that the different extracts of CF, loganin and the FH significantly shortened the immobility time in FST by 38.5%, 31.2%, 21.5%, 16.2%, 17.6%, and 39.9% (Figure 3B), respectively, and TST by 28.6%, 27.7%, 25.2%, 12.1%, 13.7%, and 30.9% (Figure 3C) compared with the control group. Furthermore, there was no significant difference in the immobility time of FST and TST between the above‐mentioned different extracts of CF. But in terms of the reversal rate, the water extract of CF had the highest reversal rate, suggesting that the its antidepressant effects is better. To exclude false positives, OFT was performed. The results showed that the different extracts of CF, loganin and the FH had no significant difference in the vertical score (Figure 3D) and horizontal score (Figure 3E) in OFT compared with the control group, suggesting that the above drugs had no locomotion‐stimulating effects in mice. These results indicated that the different extracts of CF and loganin all have antidepressant‐like actions, and the water extract of CF has better antidepressant‐like actions.

#### 3.1.3 The antidepressant‐like actions of different extracts of CF were positively correlated with the content of its main active compound loganin

To evaluate whether the antidepressant‐like actions of different extracts of CF are related to the content of loganin, this study analyzed the correlation between the reversal rate of immobility time of different extracts of CF in FST and TST and the content of loganin in different extracts. Pearson analysis showed that the reversal rate of immobility time of different extracts of CF was positively correlated with the content of loganin (Figure 3F, *r* = 0.956, for FST; Figure 3G, *r* = 0.890, for TST). These results suggested that loganin could be used as a major antidepressant active compound in CF.

### 3.2 TrkB could be used as a key target for the antidepressant‐like actions of loganin

#### 3.2.1 Loganin exerted antidepressant‐like actions in CUMS‐induced depression‐like mice

**FIGURE 4:**
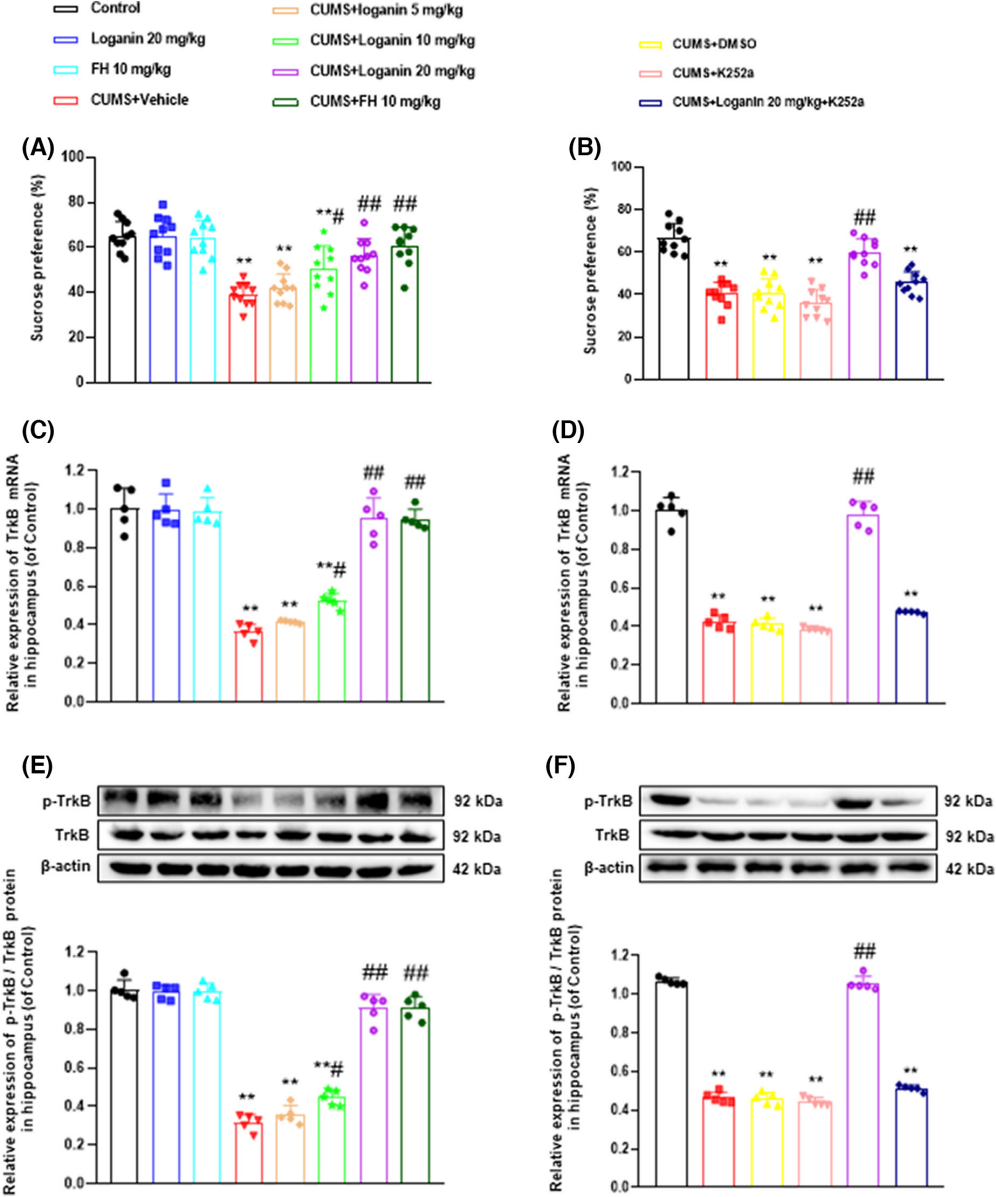
TrkB could be used as a key target for the antidepressant‐like actions of loganin. Impact of loganin (5, 10, 20 mg/kg/day; i.g for 5 weeks) on sucrose preference in SPT [*F*_(7, 72)_ = 17.711; (*p* = 0.989, *p* < 0.05, and *p* < 0.01] (A) and TrkB inhibitor K252a on sugar water preference in SPT [*F*_(5, 54)_ = 38.488; *p* < 0.01] (B), the mRNA [*F*_(7, 72)_ = 77.126, *p* = 0.945, *p* < 0.05, and *p* < 0.01] (C) and protein [*F*_(7, 72)_ = 180.020; *p* = 0.945, *p* < 0.05, and *p* < 0.01] (E) expression levels of TrkB and TrkB inhibitor K252a on the mRNA [*F*_(5, 54)_ = 223.961, *p* < 0.01] (D) and protein [*F*_(5, 54)_ = 669.062, *p* < 0.01] (F) expression levels of TrkB in the hippocampus of CUMS‐induced mice. Data on sucrose preference in SPT (*n* = 10) and the mRNA and protein expression levels of TrkB (*n* = 5) were shown as mean ± SD. *p < 0.05 and **p < 0.01 compared with the control group. ^#^ *p* < 0.05 and ^##^ *p* < 0.01 compared with the CUMS group.

On the basis of the above experiments, this study further evaluated the antidepressant‐like actions of loganin on CUMS‐induced depression‐like model. The results revealed that CUMS for 35 consecutive days significantly decreased sucrose preference in SPT (Figure 4A). Contrarily, the oral administration of loganin (10 and 20 mg/kg) and FH for 35 consecutive days significantly increased sucrose preference, while 5 mg/kg loganin did not significantly reversed. However, K252a blocked the improvement of loganin on CUMS‐induced depression‐like behavior (Figure 4B). These results showed that loganin reversed the reduction of sucrose preference in CUMS‐induced depression‐like mice in a dose‐dependent manner.

#### 3.2.2 Bloking TrkB eliminated the antidepressant‐like actions of loganin

Next, the expression level of TrkB was detected to evaluate whether TrkB could be used as a key target for loganin's antidepressant‐like actions. In the present study, the mRNA and protein expression levels of TrkB protein in the hippocampal tissues of mice was detected by quantitative real‐time polymerase chain reaction and Western blot. The results in the hippocampal tissue demonstrated that the mRNA and protein expression levels of TrkB significantly decreased in CUMS‐induced depression‐like mice (Figure 4C,E) compared with the control group. Loganin (10 and 20 mg/kg) and FH significantly reversed the CUMS‐induced decrease in the mRNA and protein expression levels of TrkB in the hippocampus, while 5 mg/kg loganin hardly reversed. However, K252a blocked the reversal effect of loganin on the mRNA and protein expression levels of TrkB (Figure 4D,F). These results suggested that TrkB might be a critical target for the antidepressant‐like actions of loganin.

### 3.3 Loganin promoted hippocampal neurotrophy and neurogenesis by regulating TrkB

#### 3.3.1 Loganin activated BDNF in hippocampus of CUMS‐induced depression‐like mice

**FIGURE 5:**
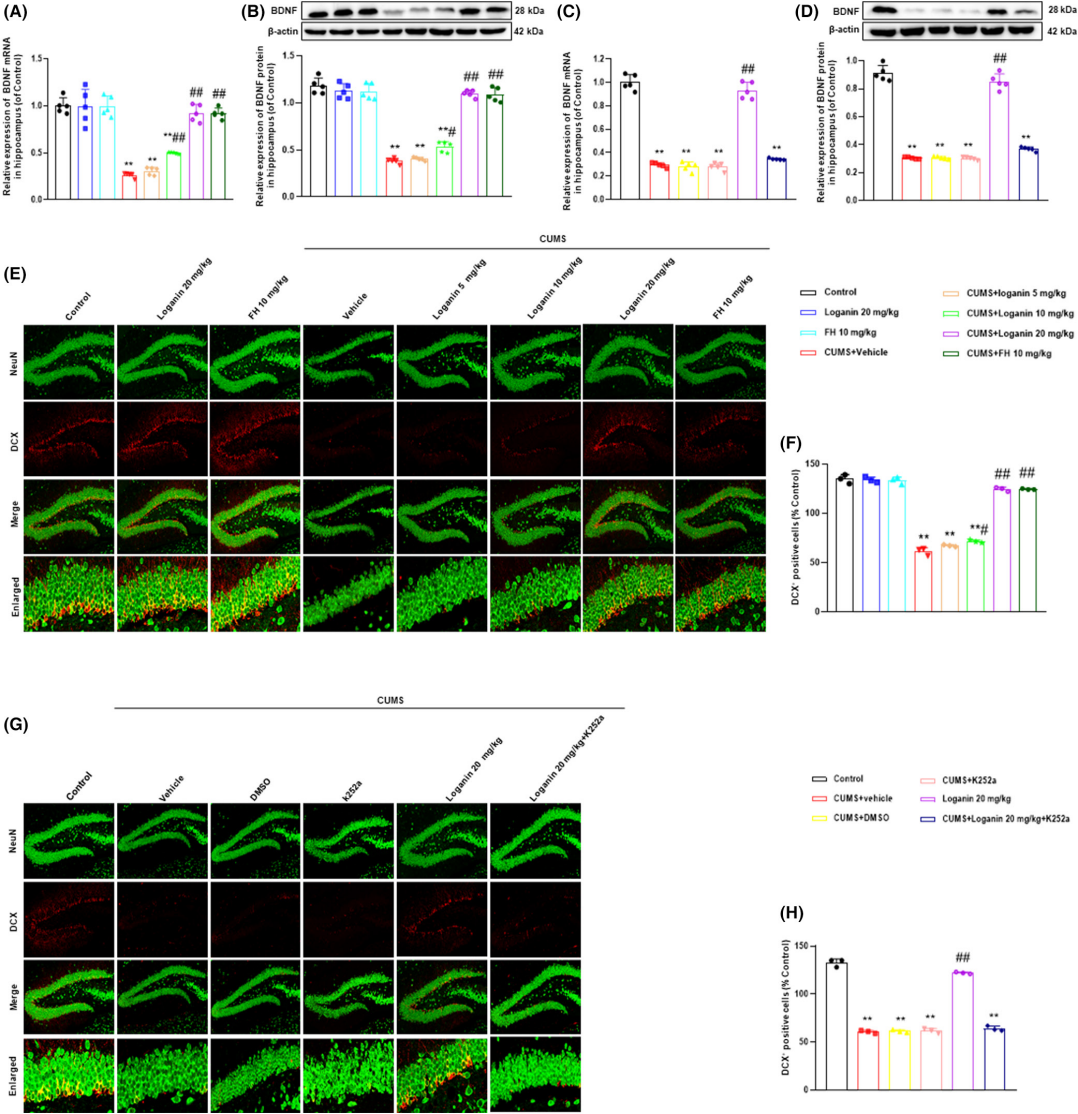
Loganin promoted hippocampal neurotrophy and neurogenesis by regulating TrkB. Impact of loganin (5, 10, 20 mg/kg/day; i.g for 35 days) on the mRNA [*F*_(7, 72)_ = 62.237, *p* = 0.995, *p* < 0.01, and *p* < 0.01] (A) and protein [*F*_(7, 72)_ = 155.811, *p* = 0.999, *p* < 0.05, and *p* < 0.01] (B) expression levels of BDNF, TrkB inhibitor K252a on the mRNA [*F*_(5, 54)_ = 281.158, *p* < 0.01] (C) and protein [*F*_(5, 54)_ = 373.912, *p* < 0.01] (D) expression levels of BDNF in the hippocampus of CUMS‐induced mice. A confocal microscopic image showed DCX (red) and NeuN (green) co‐staining (yellow) in the DG (E). The scale bar represents 50 μm for the representative images and 20 μm for the enlarged images. The number of DCX units in the DG. Density statistical analysis showed that loganin significantly increased DCX^+^ cells in the DG of CUMS‐induced mice [*F*_(7, 16)_=376.316, *p* = 0.468, *p* < 0.05, and *p* < 0.01] (F). The number of TrkB inhibitor K252a reduced DCX^+^ cells [*F*_(5, 12)_=578.783, *p* < 0.01] (G, H). Data were shown as mean ± SD (*n* = 5 or 3). *p < 0.05 and **p < 0.01 compared with the control group. ^#^ *p* < 0.05 and ^##^ *p* < 0.01 compared with the CUMS group.

Numerous studies have shown that BDNF is the most abundant neurotrophic factor in brain, which is involved in the pathophysiology and treatment of depression.^30^, ^31^, ^32^ The results in the hippocampal tissue revealed that compared with the control group, the mRNA and protein expression levels of BDNF were significantly decreased in CUMS‐induced depression‐like mice (Figure 5A,B). Loganin (10 and 20 mg/kg) and FH significantly reversed the mRNA and protein expression levels of BDNF in CUMS‐induced depression‐like mice, whereas 5 mg/kg loganin hardly reversed. In addition, K252a prevented the reversing effects of loganin on the mRNA and protein expression levels of BDNF (Figure 5C,D). These results indicated that the antidepressant mechanism of loganin involved in BDNF.

#### 3.3.2 Loganin promoted neurogenesis in hippocampus of CUMS‐induced depression‐like mice

Chronic stress not only induced depression‐like behaviors and neurotrophic deficits but also decreased neuronal proliferation and differentiation in the dentate gyrus (DG) of mice.^33^, ^34^ Therefore, we examined whether loganin treatment plays an antidepressant role by promoting hippocampal neurogenesis. The result showed that CUMS stimulation reduced the number of DCX^+^ cells in DG region compared with the control group (Figure 5E,F). In contrast, loganin (10 and 20 mg/kg) and FH significantly increased the number of DCX^+^ cells in DG, while 5 mg/kg loganin hardly reversed. However, K252a blocked the reversal effects of loganin on the levels of DCX^+^ cells in the DG region induced by CUMS (Figure 5G,H). These results indicated that loganin promoted hippocampal neurogenesis in CUMS‐induced depression‐like mice.

### 3.4 Loganin activated PI3K/Akt pathway downstream of TrkB

**FIGURE 6:**
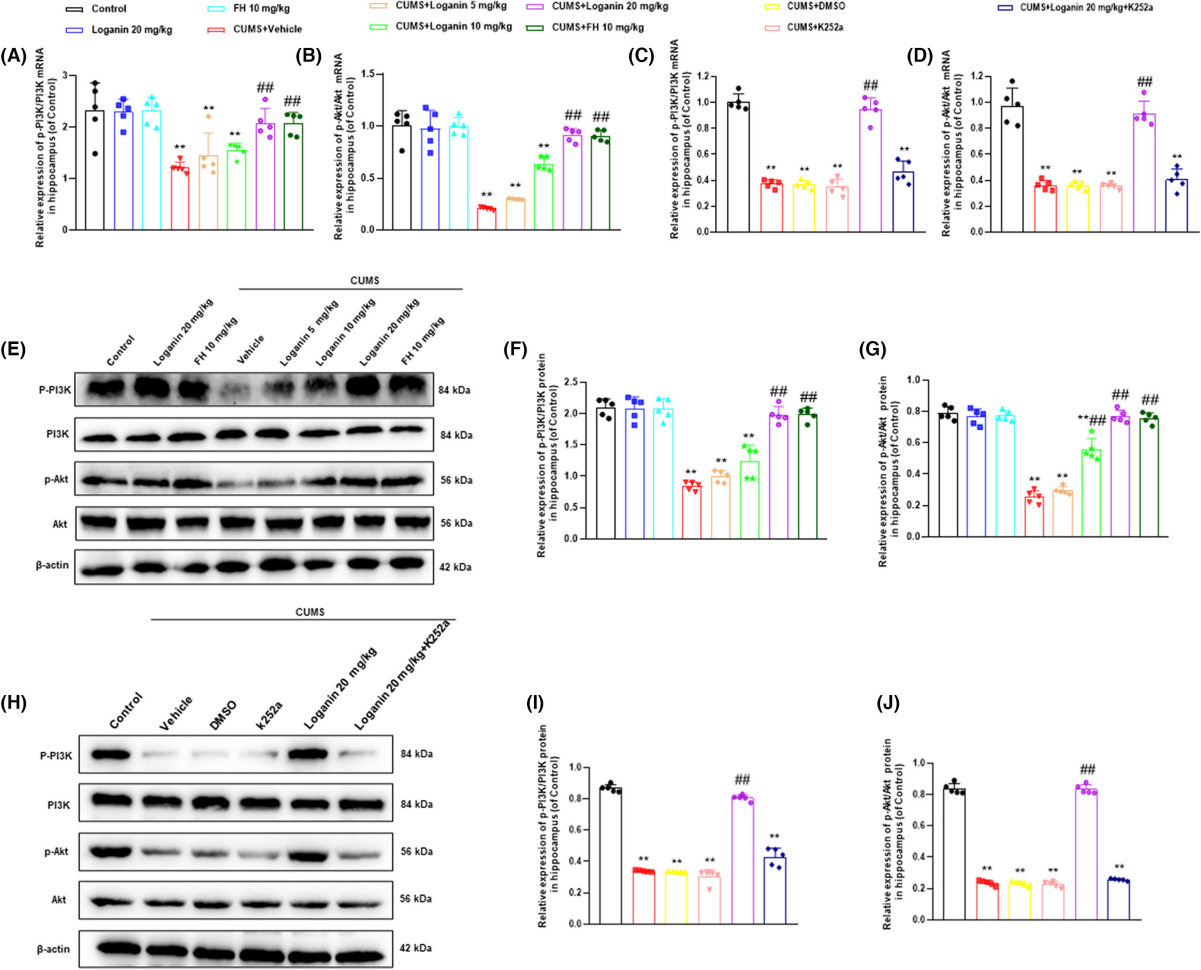
Loganin activated PI3K/Akt signal pathway downstream of TrkB. Effects of loganin (5, 10, 20 mg/kg/day; i.g for 35 days) on the mRNA [*F*_(7, 72)_ = 10.070 and 133.375; for PI3K, *p* = 0.920, *p* = 0.666, and *p* < 0.01; for Akt, *p* = 0.999, *p* = 0.271, and *p* < 0.01] (A, B) and protein [*F*_(7, 72)_ = 133.375 and 6.591; for PI3K, *p* = 1, *p* = 0.231, and *p* < 0.01; for Akt, *p* = 0.783, *p* < 0.01, and *p* < 0.01] (E, F, G) expression levels of PI3K and Akt, and TrkB inhibitor K252a on the mRNA [*F*_(5, 24)_=113.631 and 69.472, both *p* < 0.01] (C, D) and protein [*F*_(5, 24)_=372.384 and 1021.283, both *p* < 0.01] (H, I, J) expression levels of PI3K and Akt in the hippocampus of CUMS‐induced mice. Data were shown as mean ± SD (*n* = 5). *p < 0.05 and **p < 0.01 compared with the control group. ^#^ *p* < 0.05 and ^##^ *p* < 0.01 compared with the CUMS group.

Next, to investigate whether the antidepressant‐like actions of loganin is related to the TrkB‐mediated downstream PI3K/Akt pathway. The results showed that the mRNA and protein expression levels of PI3K and Akt were significantly decreased in CUMS‐induced depression‐like mice (Figure 6A,B,E–G) compared with the control group. Fortunately, 20 mg/kg loganin and FH significantly reversed the CUMS‐induced decrease in the mRNA and protein expression levels of PI3K and Akt in hippocampus, and 10 mg/kg loganin significantly reversed the abnormal expression level of Akt protein, while 5 mg/kg loganin hardly reversed. However, K252a blocked the reversing effects of loganin on the mRNA and protein expression levels of PI3K and Akt (Figure 6C,D,H–J). These results suggested that the antidepressant mechanism of loganin involved in the up‐regulation of the PI3K/Akt pathway downstream of TrkB.

### 3.5 Loganin inhibited neuroinflammation in hippocampus of CUMS‐induced depression‐like mice

**FIGURE 7:**
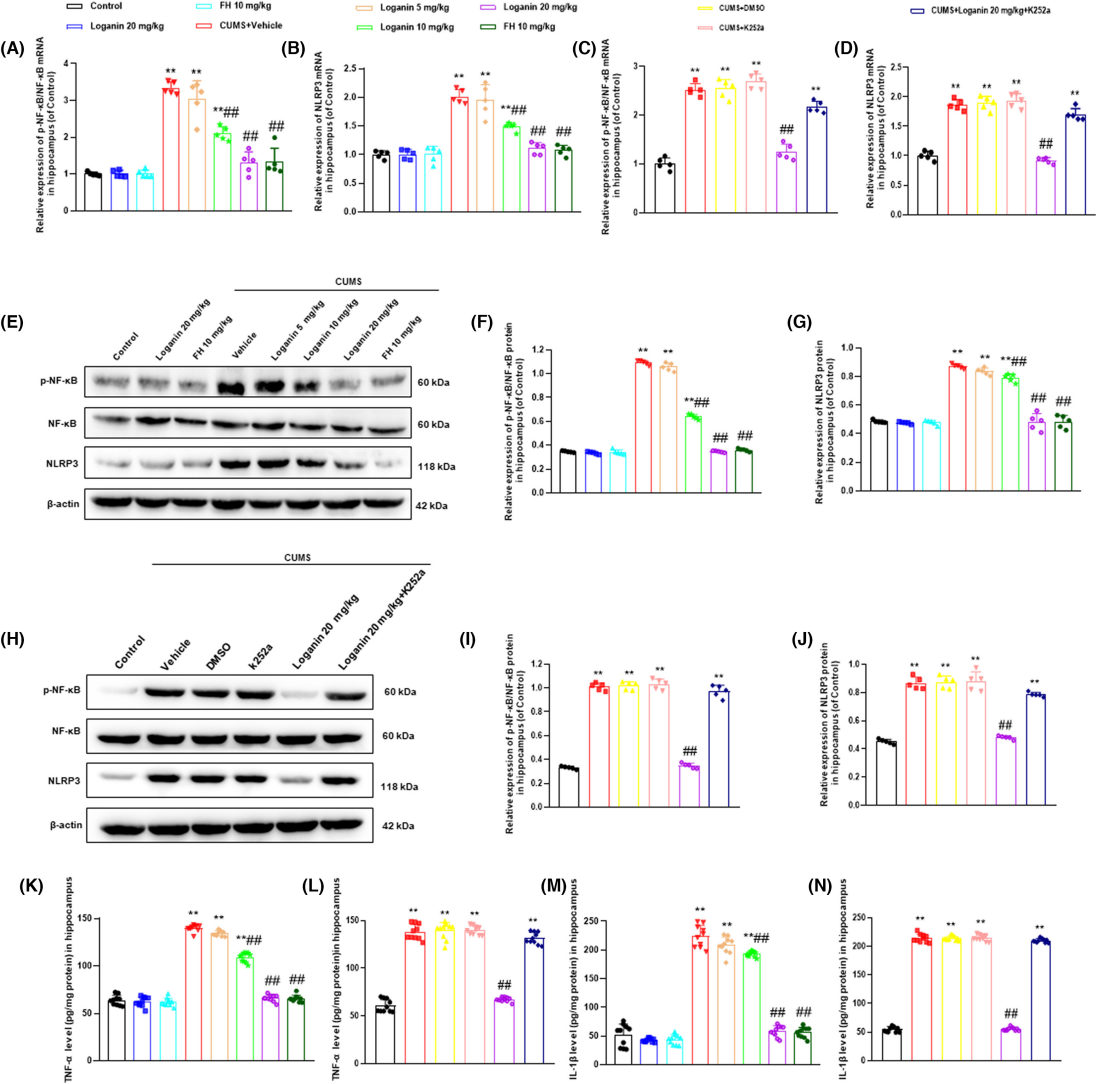
Loganin reversed the CUMS‐induced increase in hippocampal neuroinflammation related indicators. Effects of loganin (5, 10, 20 mg/kg/day; i.g for 35 days) on the mRNA [*F*_(7, 72)_ = 50.406 and 28.011; for NF‐κB, *p* = 0.544, *p* < 0.01, and *p* < 0.01; or NLRP3, *p* = 0.066, *p* < 0.01, and *p* < 0.01] (A, B) and protein [*F*_(7, 72)_ = 1633.357 and 178.399; for NF‐κB *p* = 0.999; for NLRP3, *p* = 0.591] (E, F, G) levels of NF‐κB and NLRP3, and the levels of TNF‐α [*F*_(7, 72)_ = 671.778, *p* = 0.083, *p* < 0.01, and *p* < 0.01] (K) and IL‐1β [*F*_(7, 72)_ = 439.627, *p* = 0.097, *p* < 0.01, and *p* < 0.01] (M), and TrkB inhibitor K252a on the mRNA [*F*_(5, 24)_=37.811 and 118.594, both *p* < 0.01] (C, D) and protein [*F*_(5, 24)_=496.912 and 137.637, both *p* < 0.01] (H, I, J) expression levels of NF‐κB, NLRP3, and the levels of TNF‐α [*F*_(5, 54)_ = 344.987, *p* < 0.01] (L) and IL‐1β [*F*_(5, 54)_ = 2920.046, *p* < 0.01] (N) in the hippocampus of CUMS‐induced mice. Data were shown as mean ± SD (*n* = 5 or 10). *p < 0.05 and **p < 0.01 compared with the control group. ^#^ *p* < 0.05 and ^##^ *p* < 0.01 compared with the CUMS group.

Finally, this study assessed the antidepressant‐like mechanism of loganin in terms of neuroinflammation closely related to the pathogenesis of depression.^35^ The results demonstrated that the mRNA and protein expression levels of NF‐κB and NLRP3 (Figure 7A,B,E–G) were significantly increased in CUMS‐induced depression‐like mice compared with the control group. Conversely, 20 mg/kg loganin and FH significantly reversed the CUMS‐induced increase in the mRNA and protein expression levels of NF‐κB and NLRP3 in hippocampus, whereas 5 mg/kg loganin hardly reversed. Moreover, K252a prevented the reversal effects of loganin on the mRNA and protein expression levels of NF‐κB and NLRP3 (Figure 7C,D,H–J) in hippocampus. In addition, the results revealed that compared with the control group, the levels of TNF‐α (Figure 7K) and IL‐1β (Figure 7M) were significantly increased in CUMS‐induced depression‐like mice. Loganin (10 and 20 mg/kg) and FH significantly reversed the CUMS‐induced increase in the levels of TNF‐α and IL‐1β, while 5 mg/kg loganin hardly reversed. However, K252a eliminated the inhibitory effect of loganin on the pro‐inflammatory cytokines TNF‐α (Figure 7L) and IL‐1β (Figure 7N). The above results suggested that loganin exerted antidepressant‐like actions by inhibiting neuroinflammation.

## 4 DISCUSSION

In the present study, the HPLC and behavioral despair model were used for the first time to determine that loganin could be used as a major antidepressant compound in CF. On this basis, this study for the first time confirmed that loganin plays an antidepressant‐like role by activating BDNF–TrkB signaling in the CUMS mice, and K252a was used first to discover that TrkB could be used as its antidepressant target.

Numerous studies have shown that the HPLC method was often used to determine the main active compounds of drugs.^14^, ^15^ Other studies demonstrated that behavioral despair models, including FST and TST, are widely used to screen rodent models for antidepressants.^36^, ^37^ Therefore, we evaluated the major antidepressant active compounds of CF by HPLC methods and behavioral despair models. The results showed that the content of loganin was the highest in CF water extract. Further studies showed that the different extracts of CF and 10 mg/kg loganin in FST and TST all had similar antidepressant properties to FH, especially the water extract of CF had the most obvious antidepressant properties. To exclude false positives, OFT was performed, and no locomotion‐stimulating effects were observed in mice treated with different CF extracts or loganin. In addition, Pearson analysis showed that the antidepressant‐like actions of different extracts of CF were positively correlated with the content of the loganin. These results suggested that loganin could be used as a major antidepressant active compound of CF.

As previously mentioned, clinical and preclinical studies have shown that the TrkB target was critical in the pathogenesis depression.^38^, ^39^ Therefore, after clarifying that one of the main active compounds of CF was loganin, this study further evaluated the antidepression target of loganin around TrkB. A series of studies indicated that the target of antidepressant drugs was determined by using inhibitors.^40^, ^41^ For example, previous studies showed that the antidepressant‐like actions of paeoniflorin, geniposide, and curcumin were blocked by using U0126, LY94002, and SR18292, which identified ERK, PI3K, and PGC‐1α as the targets of the above drugs, respectively.^42^, ^43^, ^44^ This study also used a similar method to diagnose whether TrkB could be used as the antidepressant target of loganin by using the recognized TrkB inhibitor K252a in CUMS‐induced depression–like mice. In this study, behavioral results displayed that 10 and 20 mg/kg loganin significantly reversed the decrease of sucrose intake in SPT induced by CUMS, while K252a blocked the antidepressant‐like actions of loganin, suggesting that TrkB might be a target for the antidepressant‐like actions of loganin. Furthermore, the results indicated thatK252a blocked the upregulation of mRNA and protein levels of TrkB in CUMS‐induced mice by loganin. These results showed that TrkB could be used as a target for the antidepressant‐like actions of loganin.

Recent evidence suggested that neurotrophy, neurogenesis, and neuroinflammation were closely related to the pathophysiology of depression.^45^, ^46^ Clinical and preclinical studies demonstrated that some antidepressants alleviated depression by activating TrkB and BDNF.^47^ Recent studies found that antidepressants enhanced neurotrophy by activating the downstream PI3K/Akt pathway of TrkB.^48^, ^49^ Moreover, numerous studies have shown that chronic stress attenuated neurogenesis, which some antidepressants were relieved via activating TrkB.^50^, ^51^ Furthermore, previous evidence shown that antidepressants inhibited neuroinflammation by activating TrkB to reduce the levels of NF‐κB, NLRP3, and pro‐inflammatory factors.^52^, ^53^ Therefore, after identifying TrkB as a key antidepressant target for loganin, we further explored its mechanism around TrkB‐mediated neurotrophy, neurogenesis, and neuroinflammatory. In the present study, loganin reversed the CUMS‐induced decrease in the mRNA and protein expression levels of BDNF, PI3K, and Akt in the hippocampus of CUMS‐induced depression‐like mice, which was blocked by TrkB antagonist K252a. Moreover, our data displayed that K252a blocked the reversal effect of loganin on the number of DCX^+^ cells in DG. Furthermore, this study showed that loganin reversed the abnormal levels of NF‐κB, NLRP3, TNF‐α, and IL‐1β in mice induced by CUMS, which was blocked by K252a. These findings demonstrated that the antidepressant‐like mechanisms of loganin involved the activation of TrkB‐mediated the enhancement of neurotrophy and neurogenesis, and the inhibition of neuroinflammation.

Taken together, loganin could be used as a major antidepressant‐like active compound in CF, and loganin exerts antidepressant‐like actions by regulating BDNF–TrkB signaling and TrkB could be used as a key target for its antidepressant‐like actions by enhancing neurotrophy and neurogenesis, and inhibiting neuroinflammation. The above findings open up new understanding of the main active compound of CF and its target and mechanisms for antidepressant‐like actions, and provide preclinical application information for its accurate and scientific application in the treatment of depression.

## AUTHOR CONTRIBUTIONS

Mingzhu Gong performed major experiments; Junming Wang performed manuscript revision; Lingling Song, Xiaohui Wu analyzed data; Yanmei Wang, Bingyin Li performed animal experiments; Yueyue Zhang reviewed the article; Lingyu Qina, Yaqian Duan performed behavior tests.

## CONFLICT OF INTEREST STATEMENT

The authors declare no conflict of interest.

## Supporting information


Appendix S1.

## Data Availability

The datasets used and/or analyzed during the current study are available from the first author upon reasonable request.

## References

[1] Moussavi S , Chatterji S , Verdes E , Tandon A , Patel V , Ustun B . Depression, chronic diseases, and decrements in health: results from the world health surveys. Lancet. 2007;370(9590):851‐858. 17826170 10.1016/S0140-6736(07)61415-9

[2] Hammen C . Risk factors for depression: an autobiographical review. Annu Rev Clin Psychol. 2018;14:1‐28. 29328780 10.1146/annurev-clinpsy-050817-084811

[3] Castrén E , Monteggia LM . Brain‐derived neurotrophic factor signaling in depression and antidepressant action. Biol Psychiatry. 2021;90(2):128‐136. 34053675 10.1016/j.biopsych.2021.05.008

[4] Joshi A , Akhtar A , Saroj P , Kuhad A , Sah SP . Antidepressant‐like effect of sodium orthovanadate in a mouse model of chronic unpredictable mild stress. Eur J Pharmacol. 2022;919:174798. 35123977 10.1016/j.ejphar.2022.174798

[5] Dwivedi Y , Rizavi HS , Zhang H , et al. Neurotrophin receptor activation and expression in human postmortem brain: effect of suicide. Biol Psychiatry. 2009;65(4):319‐328. 18930453 10.1016/j.biopsych.2008.08.035 PMC2654767

[6] Chen T , Liu S , Zheng M , Li Y , He L . The effect of geniposide on chronic unpredictable mild stress‐induced depressive mice through BTK/TLR4/NF‐κB and BDNF/TrkB signaling pathways. Phytother Res. 2021;35(2):932‐945. 33164233 10.1002/ptr.6846

[7] Jiang N , Wang H , Li C , et al. The antidepressant‐like effects of the water extract of Panax ginseng and Polygala tenuifolia are mediated via the BDNF‐TrkB signaling pathway and neurogenesis in the hippocampus. J Ethnopharmacol. 2021;267:113625. 33248184 10.1016/j.jep.2020.113625

[8] Liu SZ , Yang J , Chen LL , Wang P , Lin L . Tanshinone IIA ameliorates chronic unpredictable mild stress‐induced depression‐like behavior and cognitive impairment in rats through the BDNF/TrkB/GAT1 signaling pathway. Eur J Pharmacol. 2023;938:175385. 36379259 10.1016/j.ejphar.2022.175385

[9] Gupta GL , Sharma L , Sharma M . 18β‐Glycyrrhetinic acid ameliorates neuroinflammation linked depressive behavior instigated by chronic unpredictable mild stress via triggering BDNF/TrkB signaling pathway in rats. Neurochem Res. 2023;48(2):551‐569. 36307572 10.1007/s11064-022-03779-7 PMC9616426

[10] Yang B , Ren Q , Zhang JC , et al. Altered expression of BDNF, BDNF pro‐peptide and their precursor proBDNF in brain and liver tissues from psychiatric disorders: rethinking the brain‐liver axis. Transl Psychiatry. 2017;7(5):e1128. 28509900 10.1038/tp.2017.95 PMC5534963

[11] Yao L , Peng SX , Xu YD , et al. Unexpected neuroprotective effects of loganin on 1‐methyl‐4‐phenyl‐1,2,3,6‐tetrahydropyridine‐induced neurotoxicity and cell death in zebrafish. J Cell Biochem. 2017;118(3):615‐628. 27662601 10.1002/jcb.25749

[12] Liu J , Liu L , Tang L , et al. Protective effect and mechanism of Zuogui Jiangtang Jieyu decoction on neuron damage in hippocampal neurovascular unit in rats with autophagy‐mediated diabetes and depression. Chinese Herb Med. 2019;50:2420‐2427.

[13] Liu Q , Shi YQ , Zhang BH , et al. 2017 effect of Jieyu Anshen tablet combined with Liuwei Dihuang pill in the treatment of senile depression. J Int Psychiatry. 2017;44:85‐87, 101.

[14] Jiang N , Huang H , Wang H , et al. The antidepressant‐like effects of Shen Yuan: dependence on hippocampal BDNF–TrkB signaling activation in chronic social defeat depression‐like mice. Phytother Res. 2021;35(5):2711‐2726. 10.1002/ptr.7017 33474783

[15] Zeng J , Ji Y , Luan F , et al. Xiaoyaosan ethyl acetate fraction alleviates depression‐like behaviors in CUMS mice by promoting hippocampal neurogenesis via modulating the IGF‐1Rβ/PI3K/Akt signaling pathway. J Ethnopharmacol. 2022;288:115005. 35051601 10.1016/j.jep.2022.115005

[16] Jiang N , Huang H , Zhang Y , et al. Ginsenoside Rb1 produces antidepressant‐like effects in a chronic social defeat stress model of depression through the BDNF–Trkb signaling pathway. Front Pharmacol. 2021;12:680903. 34658847 10.3389/fphar.2021.680903 PMC8511306

[17] Zhang JJ , Gao TT , Wang Y , et al. Andrographolide exerts significant antidepressant‐like effects involving the hippocampal BDNF system in mice. Int J Neuropsychopharmacol. 2019;22(9):585‐600. 31181145 10.1093/ijnp/pyz032 PMC6754737

[18] Cui JJ , Huang ZY , Xie YH , et al. Gut microbiota mediated inflammation, neuroendocrine and neurotrophic functions involved in the antidepressant‐like effects of diosgenin in chronic restraint stress. J Affect Disord. 2023;321:242‐252. 36349650 10.1016/j.jad.2022.10.045

[19] Zhao YT , Zhang L , Yin H , et al. Hydroxytyrosol alleviates oxidative stress and neuroinflammation and enhances hippocampal neurotrophic signaling to improve stress‐induced depressive behaviors in mice. Food Funct. 2021;12(12):5478‐5487. 33998633 10.1039/d1fo00210d

[20] Zhou Y , Luo D , Shi J , et al. Loganin alleviated cognitive impairment in 3×Tg‐AD mice through promoting mitophagy mediated by optineurin. J Ethnopharmacol. 2023;312:116455. 37019163 10.1016/j.jep.2023.116455

[21] Shi R , Han Y , Yan Y , et al. Loganin exerts sedative and hypnotic effects via modulation of the serotonergic system and GABAergic neurons. Front Pharmacol. 2019;10:409. 31068813 10.3389/fphar.2019.00409 PMC6491506

[22] Tseng YT , Lin WJ , Chang WH , et al. The novel protective effects of loganin against 1‐methyl‐4‐phenylpyridinium‐induced neurotoxicity: enhancement of neurotrophic signaling, activation of IGF‐1R/GLP‐1R, and inhibition of RhoA/ROCK pathway. Phytother Res. 2019;33(3):690‐701. 30556245 10.1002/ptr.6259

[23] Liu S , Xu S , Wang Z , Guo Y , Pan W , Shen Z . Anti‐depressant‐like effect of sinomenine on chronic unpredictable mild stress‐induced depression in a mouse model. Med Sci Monit. 2018;24:7646‐7653. 30362468 10.12659/MSM.908422 PMC6215386

[24] Liu L , Dong Y , Shan X , Li L , Xia B , Wang H . Anti‐depressive effectiveness of baicalin in vitro and In vivo. Molecules. 2019;24(2):326. 30658416 10.3390/molecules24020326 PMC6359445

[25] Xu X , Piao HN , Aosai F , et al. Arctigenin protects against depression by inhibiting microglial activation and neuroinflammation via HMGB1/TLR4/NF‐κB and TNF‐α/TNFR1/NF‐κB pathways. Br J Pharmacol. 2020;177(22):5224‐5245. 32964428 10.1111/bph.15261 PMC7589024

[26] Fu H , Liu L , Tong Y , et al. The antidepressant effects of hesperidin on chronic unpredictable mild stress‐induced mice. Eur J Pharmacol. 2019;853:236‐246. 30928632 10.1016/j.ejphar.2019.03.035

[27] Zhou XM , Liu CY , Liu YY , et al. Xiaoyaosan alleviates hippocampal glutamate‐induced toxicity in the CUMS rats via NR2B and PI3K/Akt signaling pathway. Front Pharmacol. 2021;12:586788. 33912031 10.3389/fphar.2021.586788 PMC8075411

[28] Xu X , Lu YN , Cheng JH , et al. Ginsenoside Rh2 reduces depression in offspring of mice with maternal toxoplasma infection during pregnancy by inhibiting microglial activation via the HMGB1/TLR4/NF‐κB signaling pathway. J Ginseng Res. 2022;46(1):62‐70. 35035240 10.1016/j.jgr.2021.04.003 PMC8753429

[29] Li C , Xu X , Wang Z , et al. Exercise ameliorates post‐stroke depression by inhibiting PTEN elevation‐mediated upregulation of TLR4/NF‐κB/NLRP3 signaling in mice. Brain Res. 2020;1736:146777. 32171705 10.1016/j.brainres.2020.146777

[30] Zhang JC , Yao W , Hashimoto K . Brain‐derived neurotrophic factor (BDNF)‐TrkB signaling in inflammation‐related depression and potential therapeutic targets. Curr Neuropharmacol. 2016;14(7):721‐731. 26786147 10.2174/1570159X14666160119094646 PMC5050398

[31] Jia Z , Yang J , Cao Z , et al. Baicalin ameliorates chronic unpredictable mild stress‐induced depression through the BDNF/ERK/CREB signaling pathway. Behav Brain Res. 2021;414:113463. 34280458 10.1016/j.bbr.2021.113463

[32] Li K , Yan L , Zhang Y , et al. Seahorse treatment improves depression‐like behavior in mice exposed to CUMS through reducing inflammation/oxidants and restoring neurotransmitter and neurotrophin function. J Ethnophrmacol. 2020;250:112487. 10.1016/j.jep.2019.112487 31857128

[33] Gao L , Gao T , Zeng T , et al. Blockade of indoleamine 2, 3‐dioxygenase 1 ameliorates hippocampal neurogenesis and BOLD‐fMRI signals in chronic stress precipitated depression. Aging (Albany NY). 2021;13(4):5875‐5891. 33591947 10.18632/aging.202511 PMC7950278

[34] Xiao Z , Cao Z , Yang J , et al. Baicalin promotes hippocampal neurogenesis via the Wnt/β‐catenin pathway in a chronic unpredictable mild stress‐induced mouse model of depression. Biochem Pharmacol. 2021;190:114594. 33964281 10.1016/j.bcp.2021.114594

[35] Xu X , Piao HN , Aosai F , et al. Arctigenin protects against depression by inhibiting microglial activation and neuroinflammation via HMGB1/TLR4/NF‐κB and TNF‐alpha/TNFR1/NF‐κB pathways. Br J Pharmacol. 2020;177(22):5224‐5245. 32964428 10.1111/bph.15261 PMC7589024

[36] Song W , Guo Y , Jiang S , et al. Antidepressant effects of the ginsenoside metabolite compound K, assessed by behavioral despair test and chronic unpredictable mild stress model. Neurochem Res. 2018;43(7):1371‐1382. 29790069 10.1007/s11064-018-2552-5

[37] Zhang MD , Tao X , Pan RL , et al. Antidepressant‐like effects of cajaninstilbene acid and its related mechanisms in mice. Fitoterapia. 2020;141:104450. 31837410 10.1016/j.fitote.2019.104450

[38] Palmer A , Zortea M , Souza A , et al. Clinical impact of melatonin on breast cancer patients undergoing chemotherapy; effects on cognition, sleep and depressive symptoms: a randomized, double‐blind, placebo‐controlled trial. PLoS One. 2020;15(4):e231379. 10.1371/journal.pone.0231379 PMC7164654 32302347

[39] Peng Z , Zhang C , Yan L , et al. EPA is more effective than DHA to improve depression‐like behavior, glia cell dysfunction and hippcampal apoptosis signaling in a chronic stress‐induced rat model of depression. Int J Mol Sci. 2020;21(5):1769. 32150824 10.3390/ijms21051769 PMC7084382

[40] Hu Y , Zhao M , Zhao T , Qi M , Yao G , Dong Y . The protective effect of pilose antler peptide on CUMS‐induced depression through AMPK/Sirt1/NF‐κB/NLRP3‐mediated pyroptosis. Front Pharmacol. 2022;13:815413. 35401226 10.3389/fphar.2022.815413 PMC8984150

[41] Ge PY , Qu SY , Ni SJ , et al. Berberine ameliorates depression‐like behavior in CUMS mice by activating TPH1 and inhibiting IDO1‐associated with tryptophan metabolism. Phytother Res. 2022;37:342‐357. 36089660 10.1002/ptr.7616

[42] Tang M , Chen M , Li Q . Paeoniflorin ameliorates chronic stress‐induced depression‐like behavior in mice model by affecting ERK1/2 pathway. Bioengineered. 2021;12(2):11329‐11341. 34872456 10.1080/21655979.2021.2003676 PMC8810059

[43] Wang M , Yang L , Chen Z , et al. Geniposide ameliorates chronic unpredictable mild stress induced depression‐like behavior through inhibition of ceramide‐PP2A signaling via the PI3K/Akt/GSK3β axis. Psychopharmacology (Berl). 2021;238(10):2789‐2800. 34142167 10.1007/s00213-021-05895-8

[44] Wu Y , Sun F , Guo Y , et al. Curcumin relieves chronic unpredictable mild stress‐induced depression‐like behavior through the PGC‐1α/FNDC5/BDNF pathway. Behav Neurol. 2021;2021:2630413‐2630445. 10.1155/2021/2630445 PMC8692045 34950248

[45] Yao Z , Zhang Z , Zhang J , et al. Electroacupuncture alleviated the depression‐like behavior by regulating FGF2 and astrocytes in the hippocampus of rats with chronic unpredictable mild stress. Brain Res Bull. 2021;169:43‐50. 33434624 10.1016/j.brainresbull.2021.01.005

[46] Huang HJ , Chen XR , Han QQ , et al. The protective effects of ghrelin/GHSR on hippocampal neurogenesis in CUMS mice. Neuropharmacology. 2019;155:31‐43. 31103617 10.1016/j.neuropharm.2019.05.013

[47] Castrén E , Kojima M . Brain‐derived neurotrophic factor in mood disorders and antidepressant treatments. Neurobiol Dis. 2016;97:119‐126. 27425886 10.1016/j.nbd.2016.07.010

[48] Xian YF , Ip SP , Li HQ , et al. Isorhynchophylline exerts antidepressant‐like effects in mice via modulating neuroinflammation and neurotrophins: involvement of the PI3K/Akt/GSK‐3β signaling pathway. FASEB J. 2019;33(9):10393‐10408. 31233346 10.1096/fj.201802743RR

[49] Guan W , Gu JH , Ji CH , et al. Xanthoceraside administration produces significant antidepressant effects in mice through activation of the hippocampal BDNF signaling pathway. Neurosci Lett. 2021;757:135994. 34058291 10.1016/j.neulet.2021.135994

[50] Shi LS , Ji CH , Liu Y , et al. Ginsenoside Rh2 administration produces crucial antidepressant‐like effects in a CUMS‐induced mice model of depression. Brain Behav. 2022;12(8):e2705. 35848938 10.1002/brb3.2705 PMC9392527

[51] Chen XQ , Li CF , Chen SJ , et al. The antidepressant‐like effects of Chaihu Shugan san: dependent on the hippocampal BDNF‐TrkB‐ERK/Akt signaling activation in perimenopausal depression‐like rats. Biomed Pharmacothr. 2018;105:45‐52. 10.1016/j.biopha.2018.04.035 29843044

[52] Yi LT , Zhang MM , Cheng J , et al. Antidepressant‐like effects of degraded porphyran isolated from Porphyra haitanensis. Mol Nutr Food Res. 2021;65(9):e2000869. 33783973 10.1002/mnfr.202000869

[53] Dong SQ , Zhang QP , Zhu JX , et al. Gypenosides reverses depressive behavior via inhibiting hippocampal neuroinflammation. Biomed Pharmacother. 2018;106:1153‐1160. 30119182 10.1016/j.biopha.2018.07.040

