# Supplementary material for: Role of BDNF–TrkB signaling in the antidepressant‐like actions of loganin, the main active compound of Corni Fructus
Source: CNS Neurosci Ther. 2023 Jul 5;29(12):3842–53. doi: 10.1111/cns.14305 (PMC10651962; doi:10.1111/cns.14305)

Full unedited gel/blot for Figure 4E

p-TrkB

92 kDa

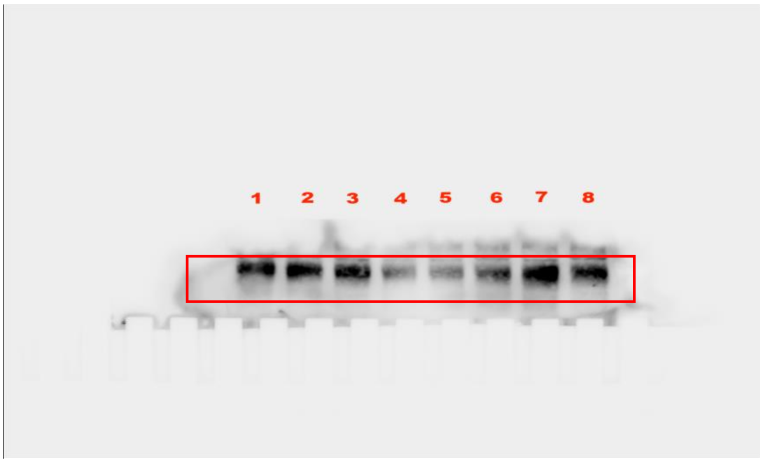

1 = Control; 2 = Loganin 20 mg/kg; 3 = FH 10 mg/kg; 4 = CUMS+Vehicle; 5 = CUMS+Loganin 5 mg/kg; 6 = CUMS+Loganin 10 mg/kg; 7 = CUMS+Loganin 20 mg/kg; 8 = CUMS+FH 10 mg/kg

TrkB

92 kDa

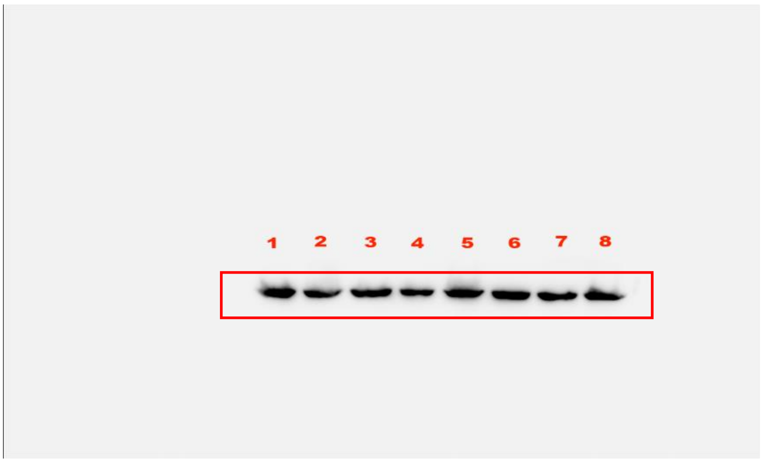

β-actin

42 kDa

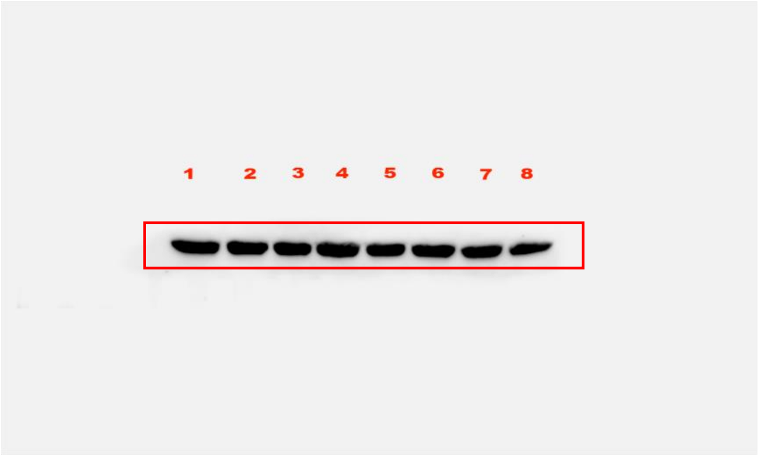

Full unedited gel/blot for Figure 4F

p-TrkB

92 kDa

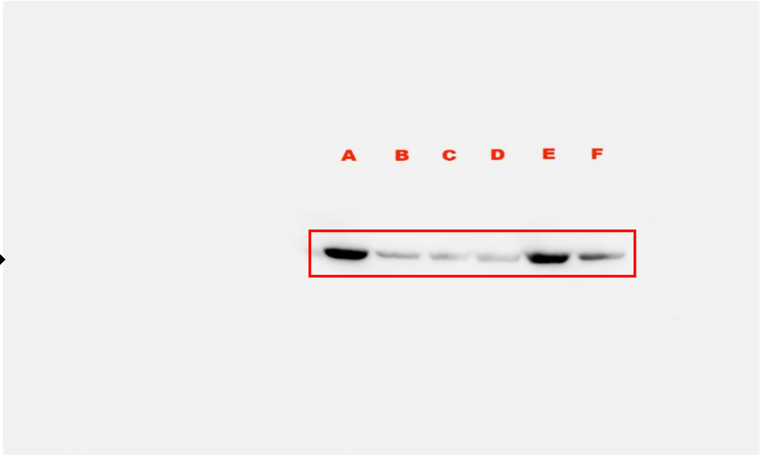

A = Control; B = CUMS+Vehicle; C = CUMS+K252a; D = CUMS+DMSO; E = CUMS+Loganin 20 mg/kg; F = CUMS+Loganin 20 mg/kg+K252a

TrkB

92 kDa

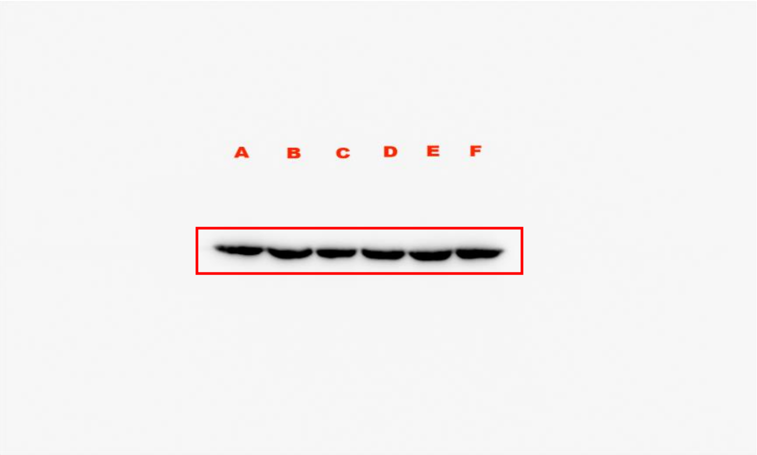

β-actin

42 kDa

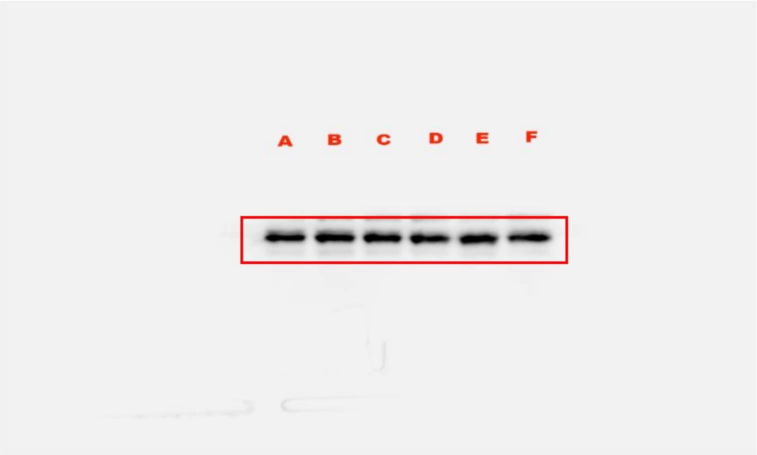

Full unedited gel/blot for Figure 5B

BDNF

28 kDa

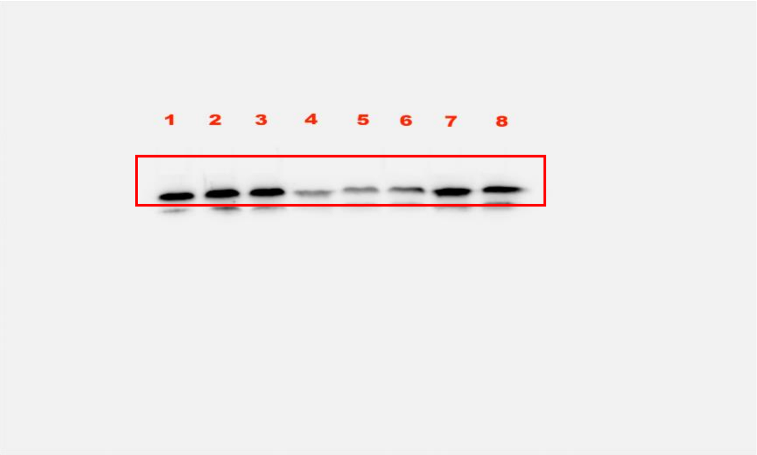

1 = Control; 2 = Loganin 20 mg/kg; 3 = FH 10 mg/kg; 4 = CUMS+Vehicle; 5 = CUMS+Loganin 5 mg/kg; 6 = CUMS+Loganin 10 mg/kg; 7 = CUMS+Loganin 20 mg/kg; 8 = CUMS+FH 10 mg/kg

β-actin

42 kDa

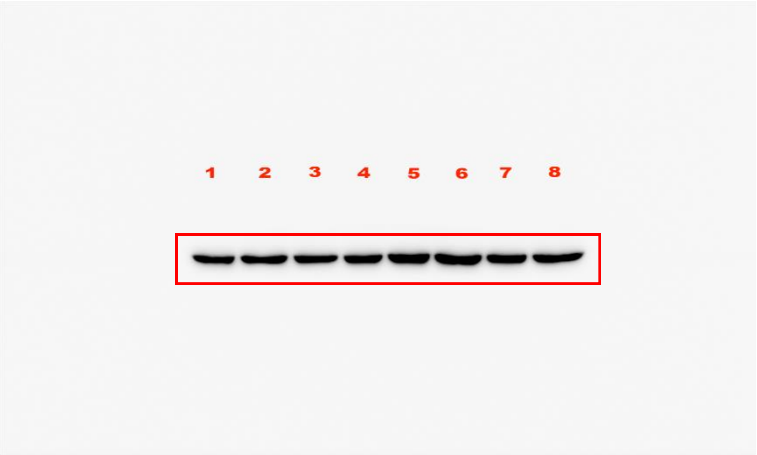

Full unedited gel/blot for Figure 5D

BDNF

28 kDa

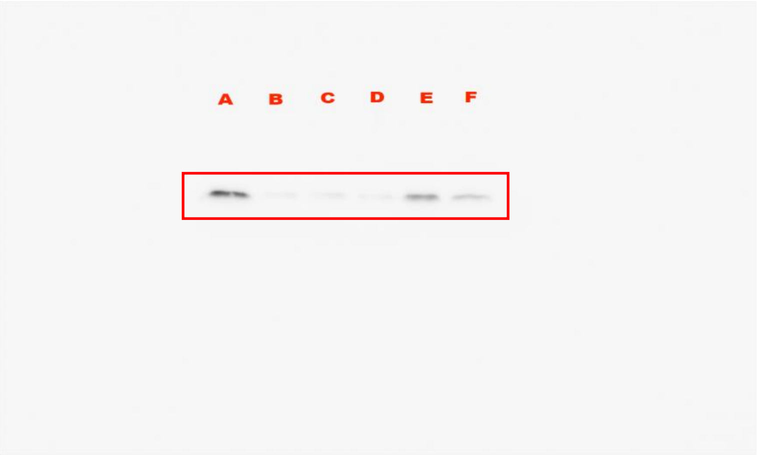

β-actin

42 kDa

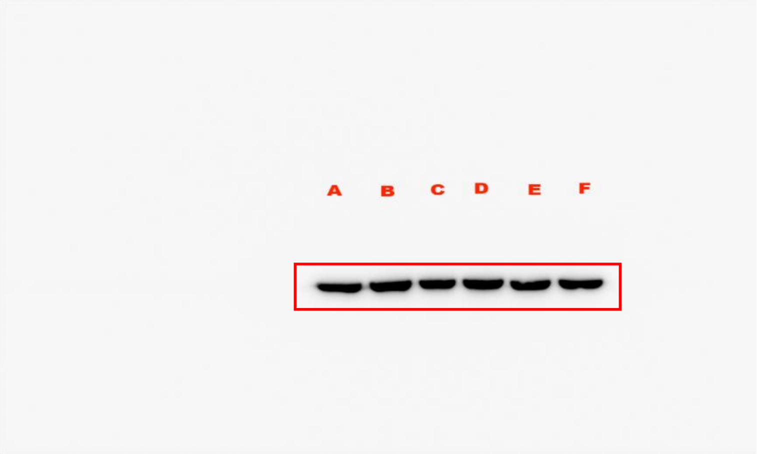

A = Control; B = CUMS+Vehicle; C = CUMS+K252a; D = CUMS+DMSO; E = CUMS+Loganin 20 mg/kg; F = CUMS+Loganin 20 mg/kg+K252a

Full unedited gel/blot for Figure 6E

p-PI3K

84 kDa

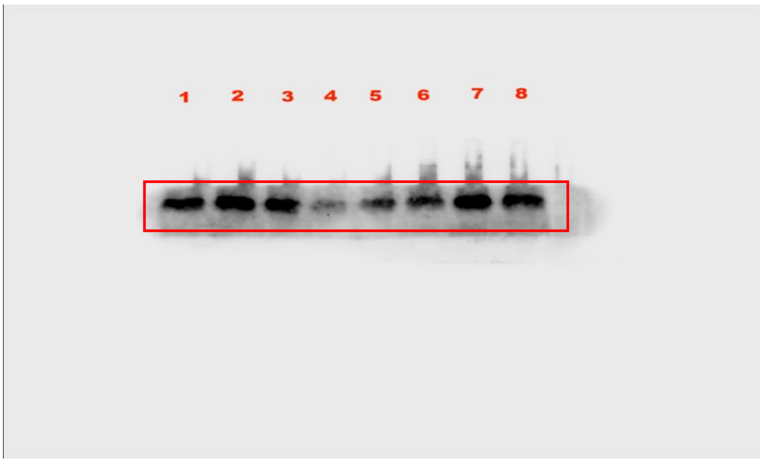

1 = Control; 2 = Loganin 20 mg/kg; 3 = FH 10 mg/kg; 4 = CUMS+Vehicle; 5 = CUMS+Loganin 5 mg/kg; 6 = CUMS+Loganin 10 mg/kg; 7 = CUMS+Loganin 20 mg/kg; 8 = CUMS+FH 10 mg/kg

PI3K

84 kDa

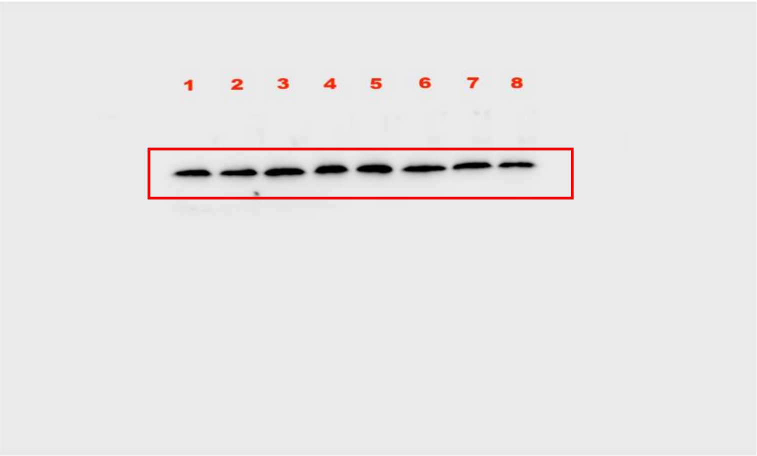

p-Akt

56 kDa

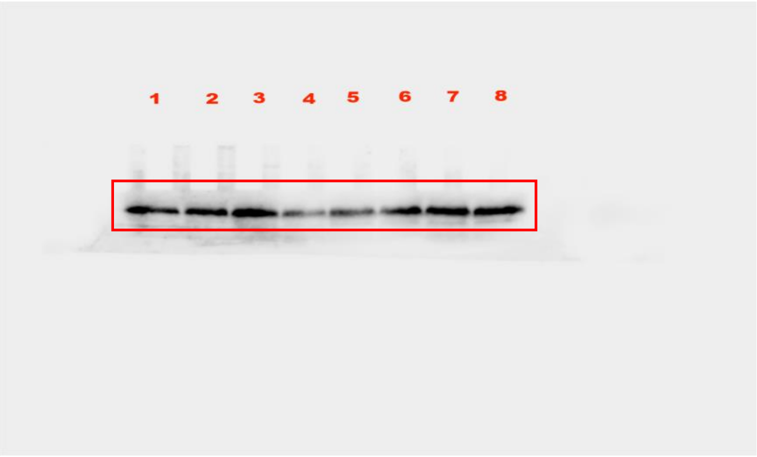

Akt

56 kDa

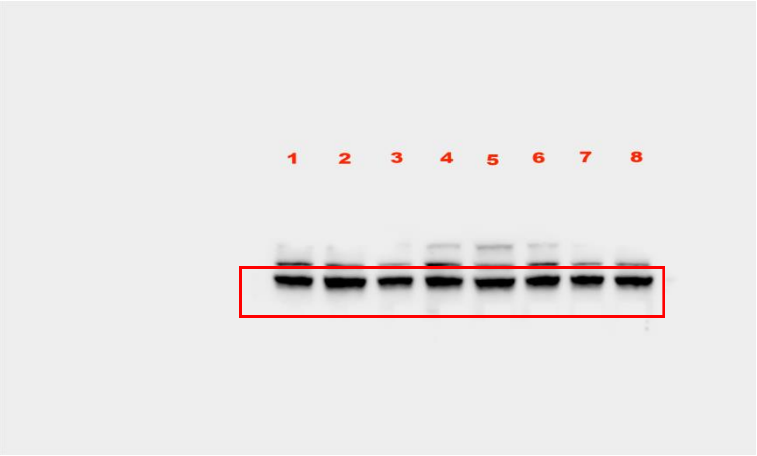

β-actin

42 kDa

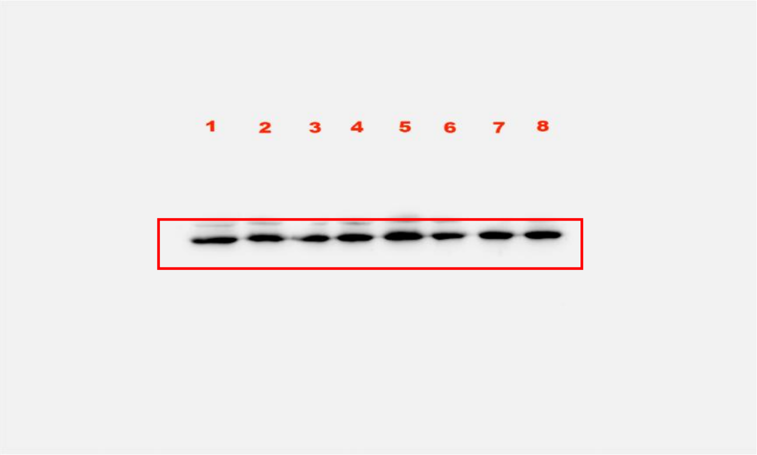

Full unedited gel/blot for Figure 6H

p-PI3K

84 kDa

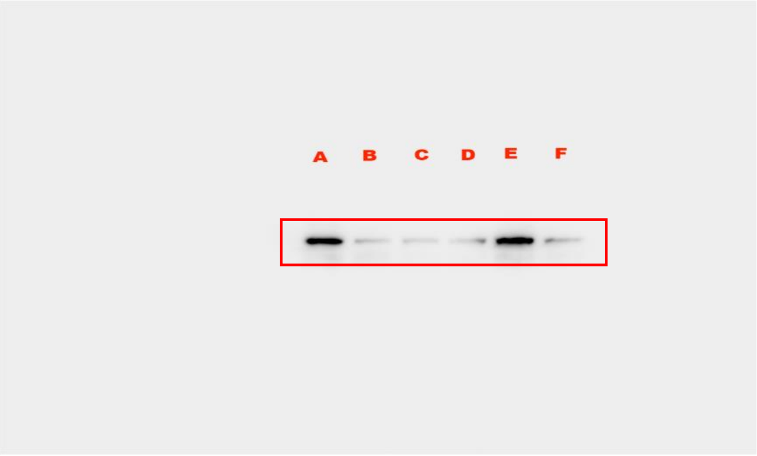

A = Control; B = CUMS+Vehicle; C = CUMS+K252a; D = CUMS+DMSO; E = CUMS+Loganin 20 mg/kg; F = CUMS+Loganin 20 mg/kg+K252a

PI3K

84 kDa

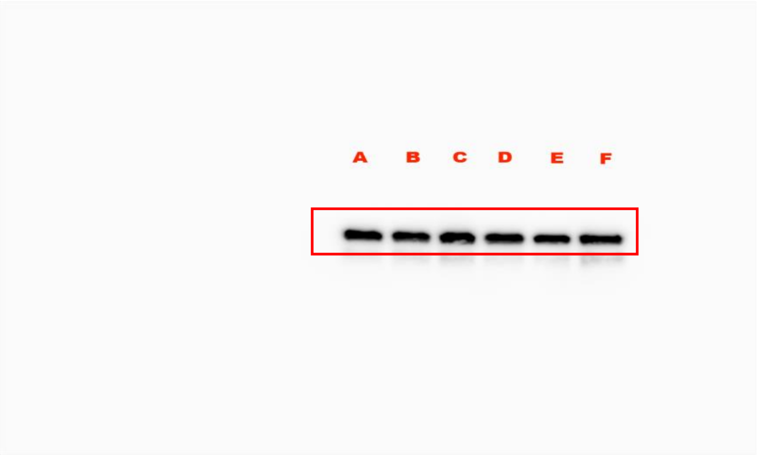

p-Akt

56 kDa

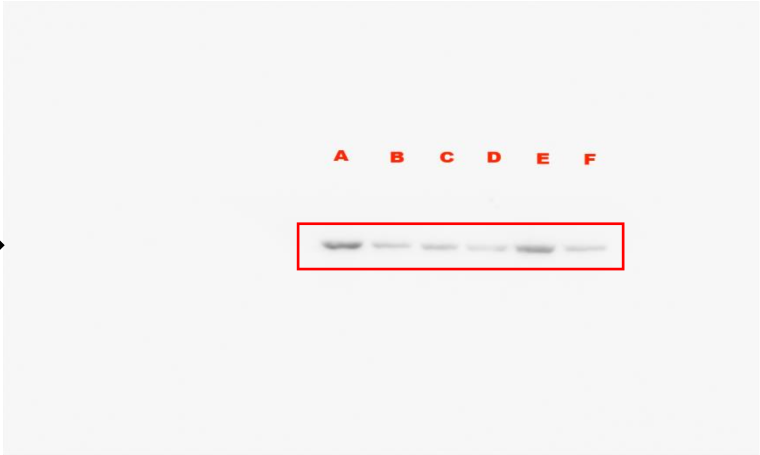

Akt

56 kDa

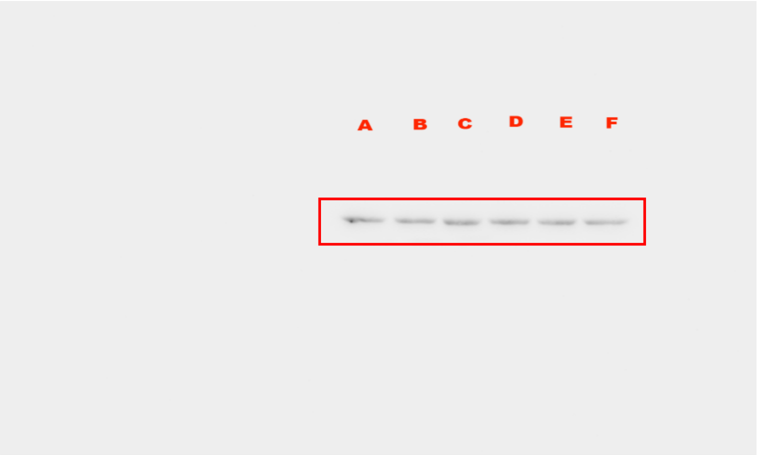

β-actin

42 kDa

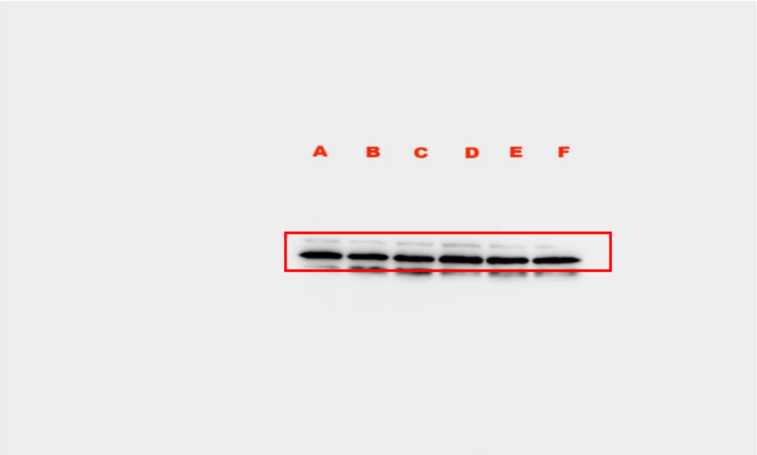

Full unedited gel/blot for Figure 7E

p-NF-κB

60 kDa

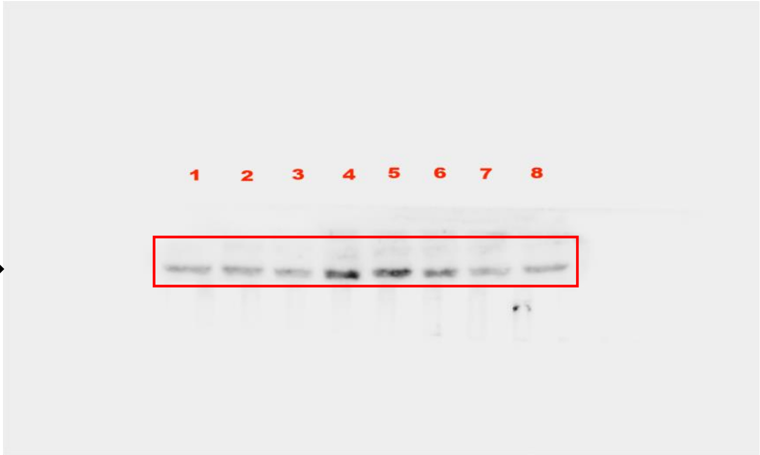

1 = Control; 2 = Loganin 20 mg/kg; 3 = FH 10 mg/kg; 4 = CUMS+Vehicle; 5 = CUMS+Loganin 5 mg/kg; 6 = CUMS+Loganin 10 mg/kg; 7 = CUMS+Loganin 20 mg/kg; 8 = CUMS+FH 10 mg/kg

NF-κB

60 kDa

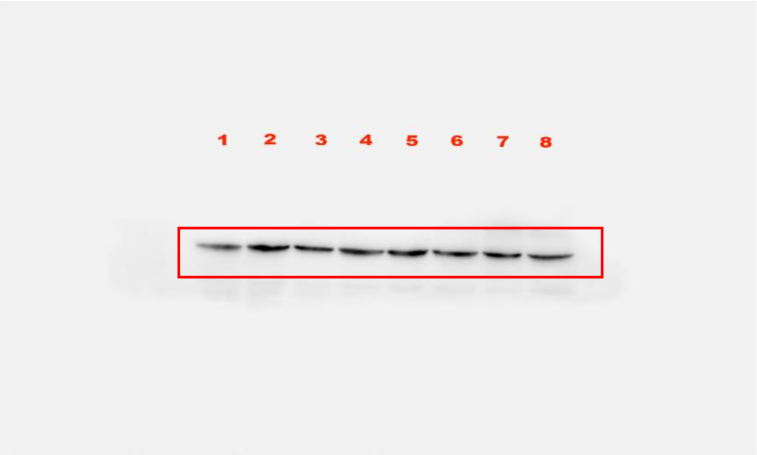

NLRP3

118 kDa

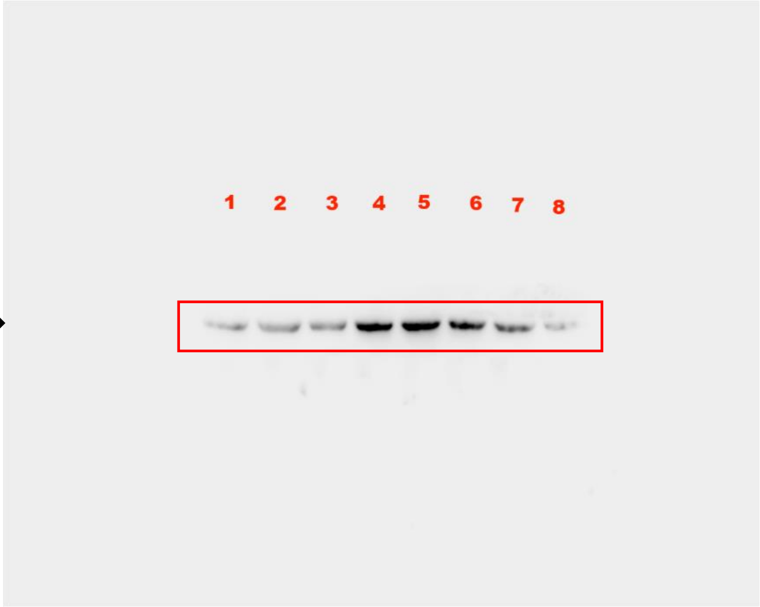

β-actin

42 kDa

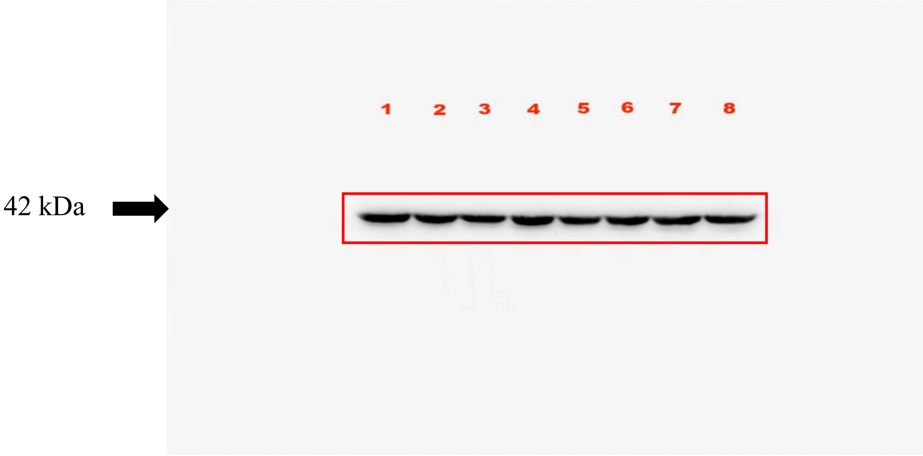

Full unedited gel/blot for Figure 7H

p-NF-κB

60 kDa

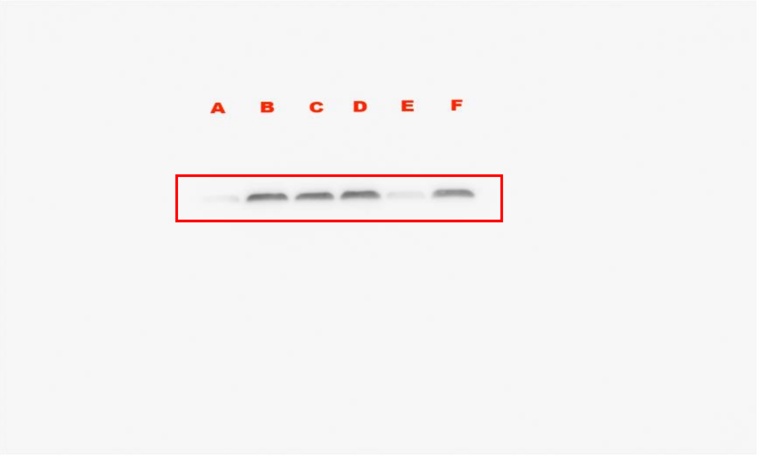

NF-κB

60 kDa

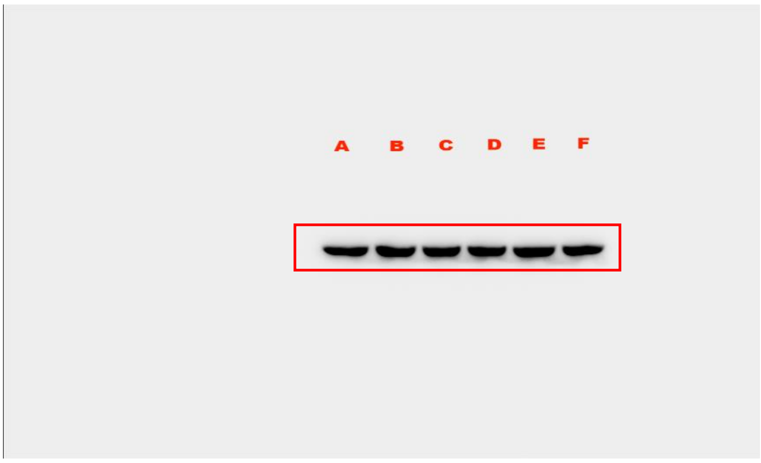

A = Control; B = CUMS+Vehicle; C = CUMS+K252a; D = CUMS+DMSO; E = CUMS+Loganin 20 mg/kg; F = CUMS+Loganin 20 mg/kg+K252a

NLRP3

118 kDa

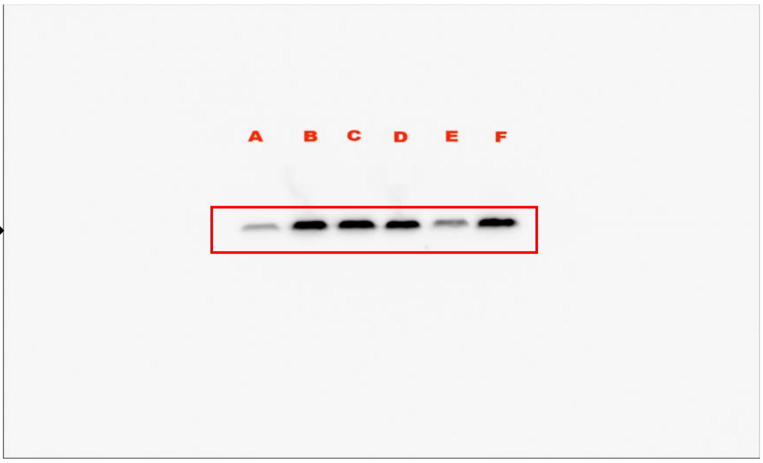

β-actin

42 kDa

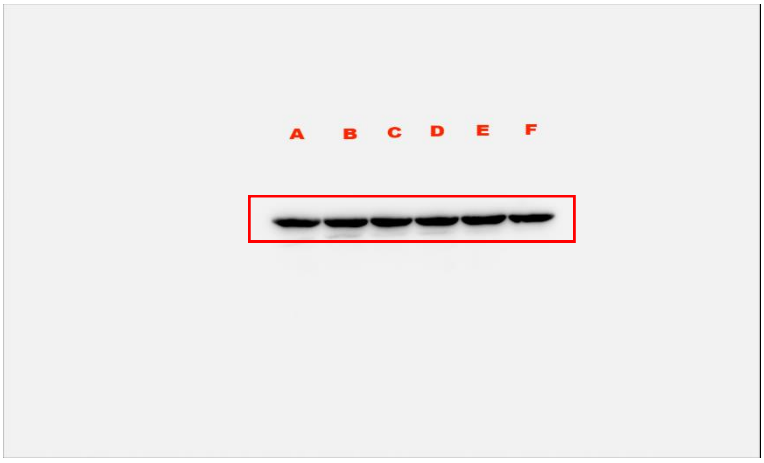

Supplement: Supplementary file 1 — Appendix S1. [file CNS-29-3842-s001.pdf]
